# Induction of Ptp2 and Cmp2 protein phosphatases is crucial for the adaptive response to ER stress in *Saccharomyces cerevisiae*

**DOI:** 10.1038/s41598-018-31413-6

**Published:** 2018-08-30

**Authors:** Tomoaki Mizuno, Meyu Nakamura, Kenji Irie

**Affiliations:** Department of Molecular Cell Biology, Faculty of Medicine, University of Tsukuba, Tsukuba, Japan

## Abstract

Expression control of the protein phosphatase is critically involved in crosstalk and feedback of the cellular signaling. In the budding yeast ER stress response, multiple signaling pathways are activated and play key roles in adaptive reactions. However, it remains unclear how the expression level of the protein phosphatase is modulated during ER stress response. Here, we show that ER stress increases expression of Ptp2 tyrosine phosphatase and Cmp2 calcineurin phosphatase. Upregulation of Ptp2 is due to transcriptional activation mediated by Mpk1 MAP kinase and Rlm1 transcription factor. This induction is important for Ptp2 to effectively downregulate the activity of Hog1 MAP kinase. The budding yeast genome possesses two genes, *CMP2* and *CNA1*, encoding the catalytic subunit of calcineurin phosphatase. *CMP2* is more important than *CNA1* not only in ER stress response, but also in salt stress response. Higher promoter activity of *CMP2* contributes to its relative functional significance in ER stress response, but is less important for salt stress response. Thus, our results suggest that expression control of Ptp2 and Cmp2 protein phosphatases at the promoter level is crucial for adaptive responses to ER stress.

## Introduction

The endoplasmic reticulum (ER) is responsible for folding and modification of nascent secretory and transmembrane proteins. When ER functions are perturbed by increased influx of newly synthesized polypeptides or exposure to stressors causing defects of glycosylation and disulfide bond formation, aberrant proteins accumulate in the ER lumen and membrane. This condition is referred to as ER stress. To restore ER homeostasis, cells under ER stress conditions activate transcription of a variety of genes, including genes encoding ER-resident chaperones and proteins functioning in the secretory pathway or ER-associated degradation^1,2^. In the budding yeast *Saccharomyces cerevisiae*, the unfolded protein response (UPR) signaling pathway composed of Ire1 and Hac1 plays a principal role in broad transcriptional response to ER stress^1,2^.

Protein phosphorylation and dephosphorylation catalyzed by protein kinases and phosphatases, respectively, are fundamental mechanisms by which cells respond to changes of the extracellular environment. Particularly, regulation of phosphorylation/dephosphorylation is essential for cells to initiate adaptive responses to environmental stresses. Previous studies using the budding yeast revealed that several kinases, including the stress responsive MAP kinases (MAPKs), such as Mpk1 and Hog1, and the Snf1 AMP-activated protein kinase (AMPK), are involved in ER stress response^3–10^. Furthermore, it has been shown that the calcineurin phosphatase participates in protection of yeast cells from ER stress^11^. It is well-known that many protein kinases can be activated through binding of their activator proteins and phosphorylation mediated by their upstream kinases. Indeed, such mechanisms operate in the budding yeast ER stress response^3–9^. In contrast, modulation of the expression levels is frequently observed in the situations where the phosphatase activity is regulated^12,13^. Furthermore, expression control of protein phosphatases is involved in feedback regulation of the signal and crosstalk between the signaling pathways^13^. In *Saccharomyces cerevisiae*, there are approximately thirty genes encoding known or putative protein phosphatases^14,15^. Previous genome-wide microarray analyses suggested that the mRNA levels of a subset of yeast protein phosphatases increase upon exposure to ER stress^16,17^. However, none of them were categorized as the target of the UPR pathway. Thus, little is known about regulation of the expression level of protein phosphatases during the budding yeast ER stress response and, furthermore, its physiological significance.

In this study, we analyzed mRNA expression patterns of 35 genes encoding yeast protein phosphatases in ER stress response. We found that the mRNA levels of 8 genes were more than two-fold increased by ER stress. Of these, *PTP2* and *CMP2* were involved in cellular sensitivity to ER stress. *PTP2* and *CMP2* encode a tyrosine phosphatase of MAPKs and a catalytic subunit of calcineurin phosphatase, respectively^18–21^. Our data presented here suggest that the *PTP2* and *CMP2* promoters are activated in response to ER stress and that promoter activation is required for the *PTP2* and *CMP2* genes to effectively fulfill their roles in ER stress response. Thus, regulation of the expression levels of protein phosphatases is critically involved in adaptive responses to ER stress.

## Results

### The mRNA levels of *PTP2* and *CMP2* are upregulated by ER stress

**Figure 1:**
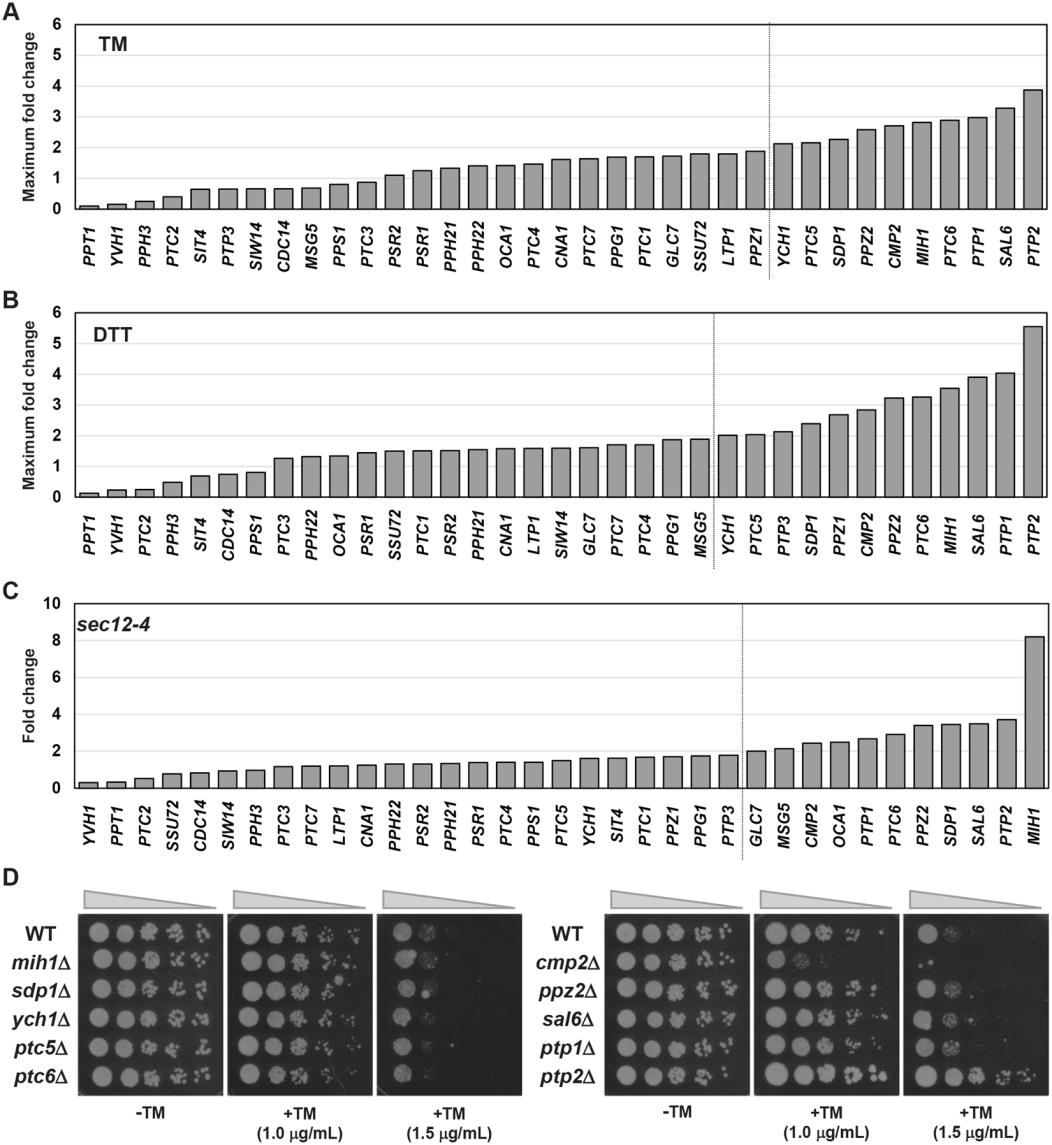
*CMP2* and *PTP2* are induced by ER stress and involved in cellular response to ER stress. (**A**,**B**) The changes of the mRNA levels of 35 genes encoding protein phosphatases after exposure to 2 μg/ml tunicamycin (TM) (**A**) and 4 mM dithiothreitol (DTT). (**B**) Maximum fold changes within 7.5 hr after ER stressor treatment relative to the untreated state are extracted from Supplementary Figs 1–10 and shown. (**C**) The changes of the mRNA levels of 35 genes encoding protein phosphatases in the *sec12-4* mutant cells. Wild-type and *sec12-4* mutant strains were grown at 25 °C until exponential phase and incubated for 4 hr at 37 °C. Fold changes in the *sec12-4* mutant cells compared to wild-type cells are extracted from Supplementary Figs 11–15 and shown. (**D**) Wild-type (WT) and *mih1*Δ, *sdp1*Δ, *ych1*Δ, *ptc5*Δ, *ptc6*Δ, *cmp2*Δ, *ppz2*Δ, *sal6*Δ, *ptp1*Δ, and *ptp2*Δ mutant strains were spotted onto YPD medium lacking or containing 1.0 or 1.5 μg/ml tunicamycin (TM) and incubated at 25 °C.

In *Saccharomyces cerevisiae*, 35 genes encode the known or putative catalytic components of protein phosphatases^14,15^ (Table 1). We first monitored their mRNA levels during ER stress response. Wild-type yeast cells were treated with tunicamycin, which causes ER stress by inhibiting N-linked glycosylation, and then harvested up to 7.5 hr every 1.5 hr; a quantitative real-time RT-PCR (qRT-PCR) analysis was carried out to quantitate the mRNA levels. We found that 10 mRNAs (*CMP2*, *MIH1*, *PPZ2*, *PTC5*, *PTC6*, *PTP1*, *PTP2*, *SAL6*, *SDP1* and *YCH1*) increased more than two-fold within 7.5 hr after tunicamycin treatment (Fig. 1A and Supplementary Figs 1–5). We performed similar experiments using dithiothreitol (DTT), which causes ER stress by inhibition of the disulfide bond formation. Exposure to DTT increased the mRNA levels of 12 genes, including *PPZ1* and *PTP3* in addition to 10 genes whose expression was increased after exposure to tunicamycin (Fig. 1B and Supplementary Figs 6–10). We next attempted to examine the mRNA levels in cells which were ER-stressed by genetic alterations. Previous reports showed that activation of the UPR pathway, which is interpreted as an indication of ER stress conditions, was caused by the block of transport form the ER to the Golgi^22–24^. The *SEC12* gene is essential for the initiation of coat protein complex II (COPII)-coated vesicle formation in ER-to-Golgi transport^25^, and its temperature-sensitive allele, *sec12-4*, causes the UPR activation^23^ (data not shown). Therefore, we analyzed expression of protein phosphatases in the *sec12-4* mutant: Wild-type and *sec12-4* mutant cells were incubated for 4 hr at 37 °C; their total RNAs were prepared and subjected to qRT-PCR analysis. The changes in gene expression patterns caused by *sec12-4* mutation closely resembled those induced by treatment with chemical ER stressors, with some exceptions such that *MIH1* expression was strongly induced by *sec12-4* mutation (Fig. 1C and Supplementary Figs 11–15). Similar results were obtained using the *sec13-1* and *sec16-2* mutants (Supplementary Figs 11–15), which are also defective in vesicle formation from the ER^23^. Remarkably, among 10 genes whose mRNAs were increased by both tunicamycin and DTT, expression of 8 genes (*CMP2*, *MIH1*, *PPZ2*, *PTC6*, *PTP1*, *PTP2*, *SAL6*, and *SDP1*) was upregulated more than two-fold in the *sec12-4*

**Table 1:** *Saccharomyces cerevisiae* genes encoding the protein phosphatase.

Family	Gene Name	ORF Name
DSP (dual specificity phosphatase)	*CDC14*	YFR028C
	*MIH1*	YMR036C
	*MSG5*	YNL053W
	*PPS1*	YBR276C
	*SDP1*	YIL113W
	*YCH1*	YGR203W
	*YVH1*	YIR026C
PPP (phosphoprotein phosphatase)	*CMP2*	YML057W
	*CNA1*	YLR433C
	*GLC7*	YER133W
	*PPG1*	YNR032W
	*PPH3*	YDR075W
	*PPH21*	YDL134C
	*PPH22*	YDL188C
	*PPT1*	YGR123C
	*PPZ1*	YML016C
	*PPZ2*	YDR436W
	*SAL6*	YPL179W
	*SIT4*	YDL047W
PPM (protein phosphatase Mg^2+^- or Mn^2+^-dependent)	*PTC1*	YDL006W
	*PTC2*	YER089C
	*PTC3*	YBL056W
	*PTC4*	YBR125C
	*PTC5*	YOR090C
	*PTC6*	YCR079W
	*PTC7*	YHR076W
PTP (protein tyrosine phosphatase)	*PTP1*	YDL230W
	*PTP2*	YOR208W
	*PTP3*	YER075C
	*LTP1*	YPR073C
	*OCA1*	YNL099C
	*SIW14*	YNL032W
Other	*PSR1*	YLL010C
	*PSR2*	YLR019W
	*SSU72*	YNL222W

mutant cells compared to wild-type cells. Thus, approximately 25% of mRNAs for protein phosphatases are upregulated under ER stress conditions.

Next, we extracted protein phosphatases that are physiologically important in ER stress response from 10 protein phosphatases that were induced by both tunicamycin and DTT. We examined ER stress sensitivity of cells deleted for each gene. Cells were plated on medium containing tunicamycin as an inducer of ER stress, and their growth was monitored. Among them, the *cmp2* mutant displayed hypersensitivity to tunicamycin, but the *ptp2* mutant was resistant to tunicamycin (Fig. 1D). The *CMP2* gene encodes a catalytic subunit of calcineurin phosphatase^18,19^. Previous studies using an inhibitor of the calcineurin phosphatase activity revealed that the budding yeast calcineurin acts to confer ER stress tolerance^11^. Furthermore, it has been reported that *ptp2* deletion results in increased resistance to ER stress^6^. Thus, our observations are consistent with previous findings.

### *PTP2* expression is induced by the Mpk1-Rlm1 signaling axis during ER stress response

**Figure 2:**
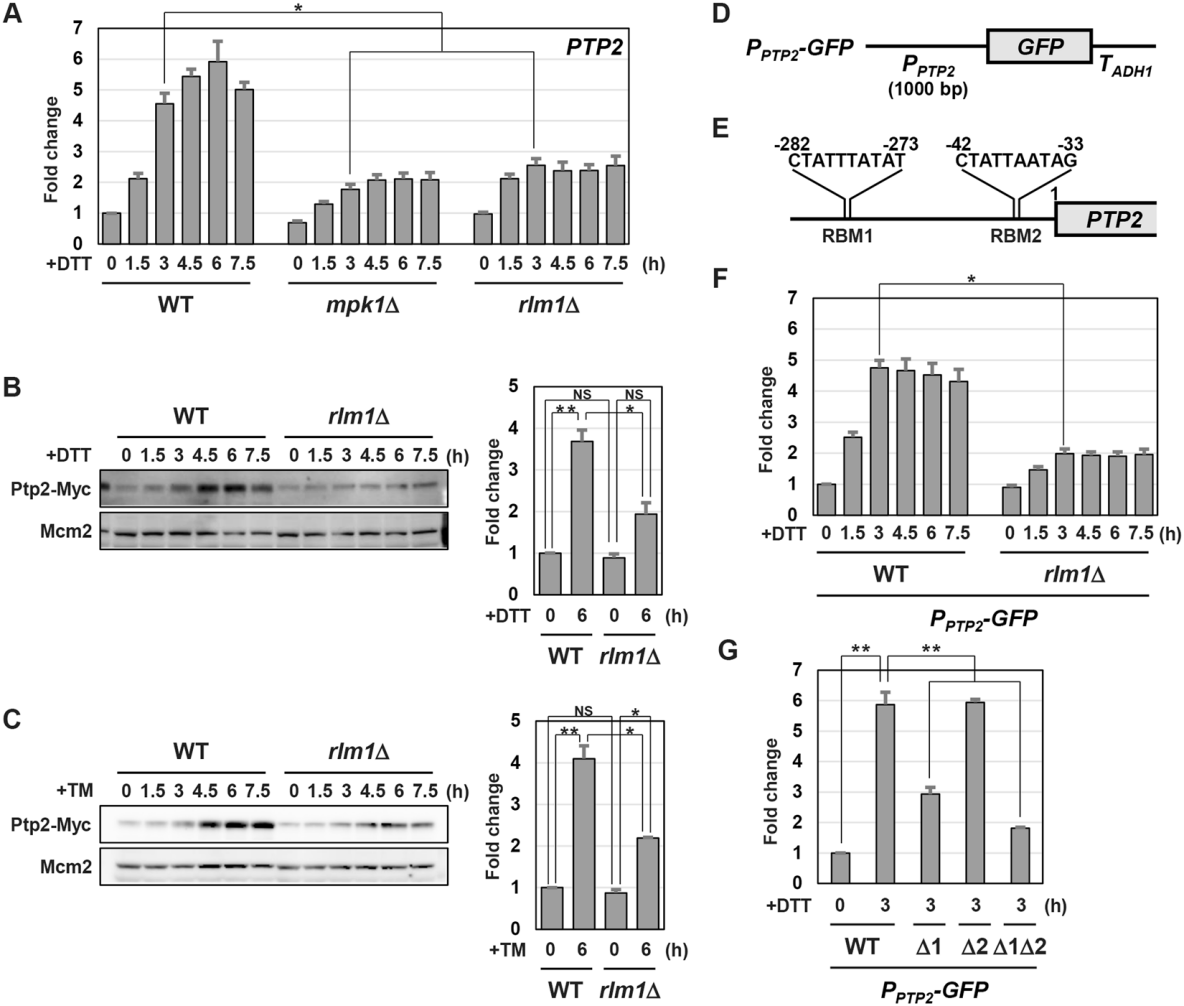
The Mpk1-Rlm1 signaling axis induces Ptp2 expression in ER stress response. (**A**) The mRNA levels of *PTP2*. Wild-type (WT) and indicated mutant strains were grown at 25 °C until exponential phase and treated with 4 mM dithiothreitol (DTT) for the indicated time, and total RNAs were prepared from each strain. The mRNA levels were quantified by qRT-PCR analysis, and relative mRNA levels were calculated using *ACT1* mRNA. The data show mean ± SEM (n = 4). *P < 0.05 as determined by Tukey’s test. (**B**,**C**) The protein levels of Ptp2. Wild-type (WT) and *rlm1* mutant strains harboring Myc-tagged *PTP2* were grown at 25 °C until exponential phase and treated with 4 mM dithiothreitol (DTT) (**B**) or 2 μg/ml tunicamycin (TM) (**C**) for the indicated time. Extracts prepared from each cell were immunoblotted with anti-Myc and anti-Mcm2 antibodies. Original blots are presented in Supplementary Figs 17 and 18. The intensities of Ptp2-Myc were measured and normalized to the Mcm2 level. The values are plotted as the fold change from wild-type cells at the time of ER stressor addition. The data show mean ± SEM (n = 3). *P < 0.05 and **P < 0.01 as determined by Tukey’s test. NS, not statistically significant (*P* > 0.05). (**D**) Schematic representation of the structure of *P*_*PTP2*_*-GFP*. (**E**) Two putative Rlm1-binding motifs in the *PTP2* promoter region. (**F**) Effects of ER stress on expression of the *P*_*PTP2*_*-GFP* reporter. Wild-type (WT) and *rlm1* mutant strains harboring the integration which expresses GFP under the control of the *PTP2* promoter were grown at 25 °C until exponential phase and treated with 4 mM dithiothreitol (DTT) for the indicated time. The *GFP* mRNA levels were quantified by qRT-PCR analysis, and relative mRNA levels were calculated using *ACT1* mRNA. The data show mean ± SEM (n = 4). *P < 0.05 as determined by Tukey’s test. (**G**) Effects of deletion mutations in putative Rlm1-binding motifs on expression of the *P*_*PTP2*_*-GFP* reporter. Wild-type (WT) cells harboring the integration which expresses GFP under the control of wild-type or mutated *PTP2* promoter were grown at 25 °C until exponential phase and treated with 4 mM dithiothreitol (DTT) for the indicated time. The *GFP* mRNA levels were quantified by qRT-PCR analysis, and relative mRNA levels were calculated using *ACT1* mRNA. The data show mean ± SEM (n = 4). **P < 0.01 as determined by Tukey’s test.

We attempted to identify the regulator of *PTP2* expression. In the budding yeast ER stress response, the UPR pathway composed of Ire1 and Hac1 activates a broad transcriptional program^1,2^. Therefore, we examined whether the UPR pathway induces expression of the *PTP2* gene. However, the mRNA levels of *PTP2* were apparently unaffected by *hac1* mutation (Supplementary Fig. 16A). Previous studies demonstrated that several signaling pathways, including Mpk1, Hog1 and Snf1, become activated in budding yeast ER stress response^3–10^. Therefore, we tested their involvement in regulation of *PTP2* expression. We found that induction of *PTP2* mRNA upon ER stress was impaired in the *mpk1* mutant, but not in *hog1* or *snf1* mutant cells (Fig. 2A, and Supplementary Fig. 16B,C). Previous studies revealed that Mpk1 phosphorylates and activates Rlm1, a transcription factor belonging to the MADS (MCM1, Agamous, Deficiens, and SRF) family^26^. Furthermore, it has been reported that Rlm1 acts downstream of Mpk1 in ER stress response^7^. We therefore examined whether Rlm1 is involved in *PTP2* upregulation during ER stress response. Similar to *mpk1* mutation, *rlm1* mutation significantly reduced the mRNA levels of *PTP2* (Fig. 2A and Supplementary Fig. 16D). We next examined the protein levels of Ptp2 during ER stress response. We used the strain expressing the carboxyl-terminally Myc-tagged Ptp2 for western blot analysis. As shown in our previous report^9^, Ptp2 was increased following exposure to ER stressors (Fig. 2B,C). However, Ptp2 induction was diminished by *mpk1* and *rlm1* mutations (Fig. 2B,C, and Supplementary Fig. 16E). These results indicate that ER stress-activated Mpk1-Rlm1 pathway upregulates the expression level of the *PTP2* gene.

Previous reports showed that *PTP2* expression is induced by heat shock in a manner dependent on Mpk1 and Rlm1^27,28^. Hence, it has been assumed that Rlm1 transcriptionally activates the *PTP2* gene in response to heat shock; however, it remains unclear whether the *PTP2* promoter is really activated. Therefore, we investigated whether ER stress increases *PTP2* promoter activity. To test this, we constructed the *P*_*PTP2*_*-GFP* reporter (Fig. 2D), consisting of the 5′ upstream region of the *PTP2* gene to drive *GFP* expression, and monitored the mRNA levels of *GFP* by qRT-PCR (Fig. 2F). *GFP* expression from the *P*_*PTP2*_*-GFP* reporter was increased after treatment with ER stressors (Fig. 2F and Supplementary Fig. 16F). However, *GFP* induction was impaired by *rlm1* mutation (Fig. 2F). This suggests that the *PTP2* promoter is activated by ER stress in a manner dependent on Rlm1. We further explored the mechanism by which Rlm1 controls *PTP2* promoter activity. Previous analysis revealed that Rlm1 binds the consensus sequence, CTA(T/A)(T/A)(T/A)(T/A)TAG^29^. Our sequence analysis utilizing JASPAR, a database of transcription factor binding profiles (http://jaspar.genereg.net/), showed that two putative binding motifs for Rlm1 exist in the 5′ upstream region of the *PTP2* gene (Fig. 2E). We designated them as the Rlm1-binding motifs (RBMs). To examine whether the RBMs are really important for *PTP2* induction during ER stress response, we deleted each RBM or both in the *P*_*PTP2*_*-GFP* reporter. Deletion of RBM1, the sequence spanning from −282 to −273, clearly suppressed *GFP* induction (Fig. 2G). On the other hand, deletion of RBM2, the sequence spanning from −42 to −33, alone failed to change *GFP* expression pattern, but inhibited *GFP* upregulation in combination to RBM1 deletion (Fig. 2G and Supplementary Fig. 16F). This result supports the model whereby Rlm1 transcriptionally activates the *PTP2* gene during ER stress response.

### Ptp2 induction is required for effective downregulation of Hog1 activity in ER stress response

**Figure 3:**
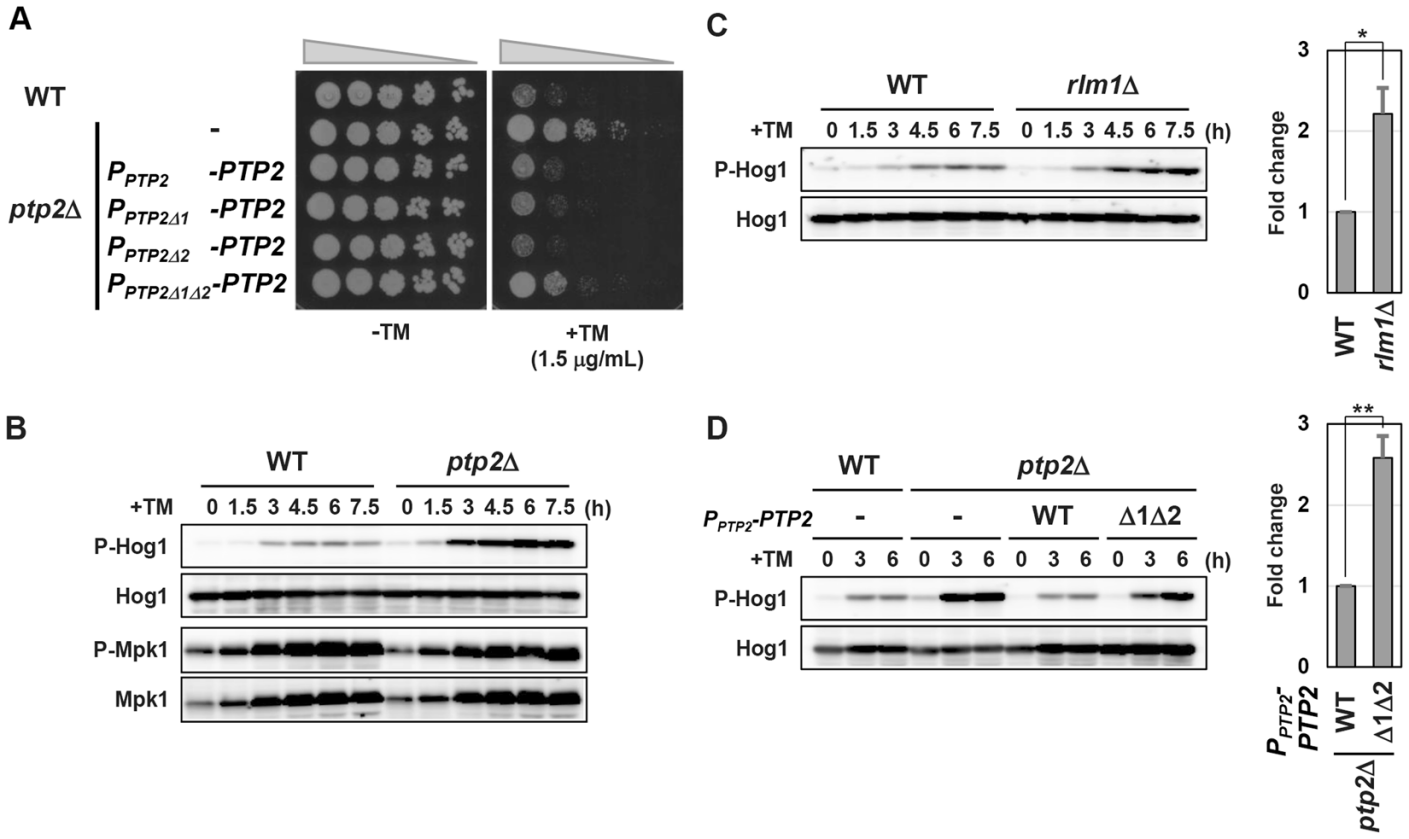
Ptp2 induction is required for effective downregulation of Hog1 activity. (**A**) Effects of the *P*_*PTP2*_*-PTP2* integrations on ER stress sensitivity. Wild-type (WT) and *ptp2* mutant strains harboring the *P*_*PTP2*_*-PTP2* integrations were spotted onto YPD medium lacking or containing 1.5 μg/ml tunicamycin (TM) and incubated at 25 °C. (**B**) Effects of *ptp2*Δ mutation on ER stress-induced Hog1 and Mpk1 activation. Wild-type (WT) and indicated mutant strains were grown at 25 °C until exponential phase and treated with 2 μg/ml tunicamycin (TM) for the indicated time. Extracts prepared from each cell were immunoblotted with anti-phospho-p38 MAPK (P-Hog1), anti-Hog1, anti-phospho-p44/42 MAPK (P-Mpk1), anti-Mpk1 antibodies. Original blots are presented in Supplementary Fig. 19. (**C**) Effects of *rlm1*Δ mutation on ER stress-induced Hog1 activation. Wild-type (WT) and the *rlm1*Δ mutant strains were grown at 25 °C until exponential phase and treated with 2 μg/ml tunicamycin (TM) for the indicated time. Extracts prepared from each cell were immunoblotted with anti-phospho-p38 MAPK (P-Hog1) and anti-Hog1 antibodies. Original blots are presented in Supplementary Fig. 20. The intensities of phosphorylated Hog1 were measured and normalized to total Hog1 level. The values are plotted as the fold change from wild-type cells at 6 hr after tunicamycin addition. The data show mean ± SEM (n = 3). *P < 0.05 as determined by Student’s *t*-test. (**D**) Effects of the *P*_*PTP2*_*-PTP2* integrations on ER stress-induced Hog1 activation. Wild-type (WT) and *ptp2* mutant strains harboring the *P*_*PTP2*_*-PTP2* integrations were grown at 25 °C until exponential phase and treated with 2 μg/ml tunicamycin (TM) for the indicated time. Extracts prepared from each cell were immunoblotted with anti-phospho-p38 MAPK (P-Hog1) and anti-Hog1 antibodies. Original blots are presented in Supplementary Fig. 21. The intensities of phosphorylated Hog1 were measured and normalized to total Hog1 level. The values are plotted as the fold change from the *ptp2* mutant cells harboring wild-type *P*_*PTP2*_*-PTP2* integration at 6 hr after tunicamycin addition. The data show mean ± SEM (n = 3). **P < 0.01 as determined by Student’s *t*-test.

To investigate whether ER stress-induced upregulation of Ptp2 is critical for its role in ER stress response, we examined ER stress sensitivity of cells in which *PTP2* induction is impaired. The recent study revealed that Mpk1 is involved in the ER stress surveillance (ERSU) pathway, which prevents transmission of stressed ER into daughter cells, and the defect in ERSU causes hypersensitivity to ER stress^7^. Rlm1 has been reported to act downstream of Mpk1 in ER stress response^4,7^. Indeed, hypersensitivity to ER stress was observed in the *rlm1* mutant^4^ (Supplementary Fig. 16G). Thus, we considered that the *mpk1* and *rlm1* mutants are not suitable to be used as cells with diminished *PTP2* induction. Therefore, we generated a series of *P*_*PTP2*_*-PTP2* constructs, which express *PTP2* under the control of wild-type or mutated *PTP2* promoter, and compared their ability to complement the ER stress resistant phenotype caused by *ptp2* mutation (Fig. 3A). The *ptp2* mutant was resistant to ER stress. When harboring wild-type *P*_*PTP2*_*-PTP2* integration, ER stress sensitivity of the *ptp2* mutants was comparable to wild-type cells. Deletion of each RBM had little effect on the ability of *P*_*PTP2*_*-PTP2* integration to complement the *ptp2* mutant phenotype. However, the *P*_*PTP2*_*-PTP2* integration in which both RBMs were deleted only partly complement ER stress resistance caused by *ptp2* mutation. These results suggest that ER stress-induced Ptp2 upregulation is important for its role in ER stress response.

Ptp2 is known to dephosphorylate and inactivate Hog1 MAPK^20,21^. A previous report showed that loss of Ptp2 causes enhanced ER stress resistance in a *HOG1*-dependent manner^6^. This phenomenon was seen in our strain background (data not shown). On the other hand, it has also been proposed that Ptp2 functions in dephosphorylation and inactivation of Mpk1^27,28^. Therefore, we examined the effects of *ptp2* mutation on the activities of Hog1 and Mpk1 during ER stress response. It is well-known that antibodies against the phosphorylated form of mammalian p38 and p42/p44 MAPKs can be utilized to detect the phosphorylated form of the budding yeast Hog1 and Mpk1, respectively. We therefore performed western blot analysis using anti-phospho-p38 antibodies to monitor Hog1 activity. As observed previously^5,9^, tunicamycin treatment increased Hog1 activity in wild-type cells (Fig. 3B). In the *ptp2* mutant cells, tunicamycin-induced Hog1 activation was significantly enhanced (Fig. 3B), indicating that Ptp2 dephosphorylates and inactivates Hog1 in ER stress response. We next determined Mpk1 activity by western blot analysis using anti-phospho-p42/p44 antibodies. Consistent with previous observations^4,7^, Mpk1 became activated by tunicamycin exposure (Fig. 3B). The activated Mpk1 level in the *ptp2* mutant was comparable to that in wild-type cells (Fig. 3B). Western blot analysis also showed that the amount of Mpk1 was increased after ER stress treatment (Fig. 3B). This increase has been reported to result from Rlm1-mediated transcriptional activation of the *MPK1* gene^7^. Similar to wild-type cells, Mpk1 upregulation was observed in the *ptp2* mutants, suggesting that Ptp2 is not involved in regulation of the Mpk1-Rlm1 pathway in ER stress response. Thus, Ptp2 acts as a negative regulator of Hog1 in ER stress response.

We next examined how Rlm1-mediated transcriptional activation of the *PTP2* gene influences Hog1 activity in ER stress response. Hog1 activation upon tunicamycin exposure was upregulated in the *rlm1* mutant (Fig. 3C). This result suggests that Rlm1 is involved in downregulation of Hog1 activity during ER stress response. To test whether that ER stress-induced activation of the *PTP2* promoter is important for Hog1 downregulation, we compared the Hog1 activity in the *ptp2* mutant cells harboring *P*_*PTP2*_*-PTP2* integrations (Fig. 3D). When carrying wild-type *P*_*PTP2*_*-PTP2* integration, the *ptp2* mutant displayed Hog1 activity at comparable levels to wild-type cells. However, Hog1 activation was upregulated by deleting both RBMs from the *P*_*PTP2*_*-PTP2* integration. Taken together, these results indicate that Rlm1-mediated transcriptional activation of the *PTP2* gene is critically involved in Hog1 downregulation during ER stress response.

### The *CMP2* promoter is important for ER stress response, but not for salt stress response

**Figure 4:**
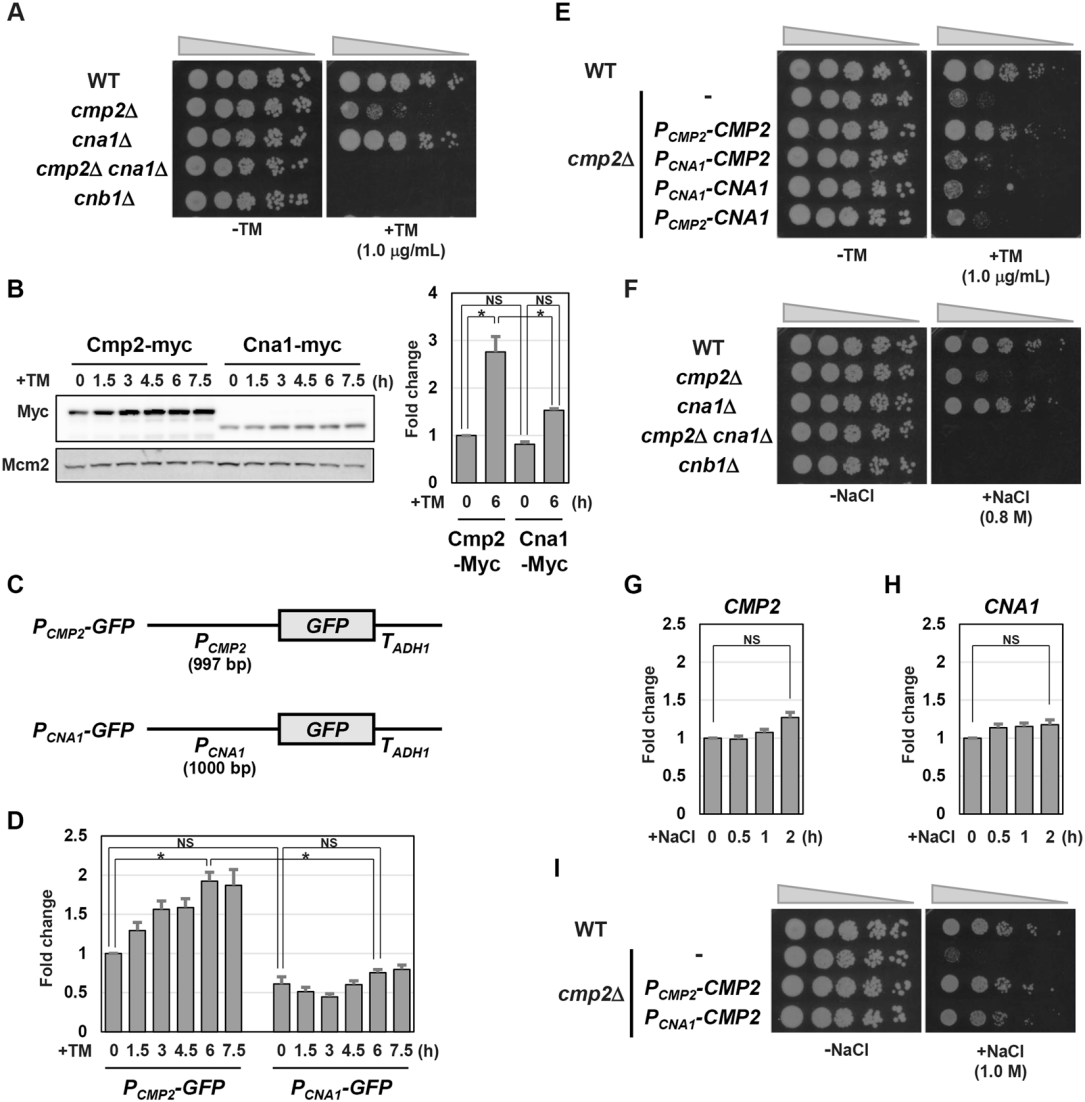
Cmp2 induction mediated by the promoter activation is important for ER stress response. (**A**) ER stress sensitivity of the mutants in calcineurin subunits. Wild-type (WT) and *cmp2*Δ, *cna1*Δ, *cmp2*Δ *cna1*Δ, and *cna1*Δ mutant strains were spotted onto YPD medium lacking or containing 1.0 μg/ml tunicamycin (TM) and incubated at 25 °C. (**B**) The protein levels of Cmp2 and Cna1. Wild-type (WT) cells harboring Myc-tagged *CMP2* or *CNA1* were grown at 25 °C until exponential phase and treated with 2 μg/ml tunicamycin (TM) for the indicated time. Extracts prepared from each cell were immunoblotted with anti-Myc and anti-Mcm2 antibodies. Original blots are presented in Supplementary Fig. 22. The intensities of Cmp2-Myc and Cna1-Myc were measured and normalized to the Mcm2 level. The values are plotted as the fold change from the level of Cmp2 at the time of tunicamycin addition. The data show mean ± SEM (n = 3). *P < 0.05 as determined by Tukey’s test. NS, not statistically significant (*P* > 0.05). (**C**) Schematic representation of the structure of *P*_*CMP2*_*-GFP* and *P*_*CNA1*_*-GFP*. (**D**) Effects of ER stress on expression of the *P*_*CMP2*_*-GFP* and *P*_*CNA1*_*-GFP* reporters. Wild-type (WT) cells harboring the integration which expresses GFP under the control of the *CMP2* or *CNA1* promoter were grown at 25 °C until exponential phase and treated with 2 μg/ml tunicamycin (TM) for the indicated time. The *GFP* mRNA levels were quantified by qRT-PCR analysis, and relative mRNA levels were calculated using *ACT1* mRNA. The data show mean ± SEM (n = 4). *P < 0.05 as determined by Tukey’s test. NS, not statistically significant (*P* > 0.05). (**E**) Effects of *P*_*CMP2*_*-CMP2*, *P*_*CNA1*_*-CMP2*, *P*_*CNA1*_*-CNA1*, or *P*_*CMP2*_*-CNA1* integrations on ER stress sensitivity. Wild-type (WT) and *cmp2* mutant strains harboring the integration of *P*_*CMP2*_*-CMP2*, *P*_*CNA1*_*-CMP2*, *P*_*CNA1*_*-CNA1*, or *P*_*CMP2*_*-CNA1* were spotted onto YPD medium lacking or containing 1.0 μg/ml tunicamycin (TM) and incubated at 25 °C. (**F**) Salt sensitivity of the mutants in calcineurin subunits. Wild-type (WT) and *cmp2*Δ, *cna1*Δ, *cmp2*Δ *cna1*Δ, and *cna1*Δ mutant strains were spotted onto YPD medium lacking or containing 0.8 M sodium chloride (NaCl) and incubated at 25 °C. (**G**,**H**) The mRNA levels of *CMP2* (**G**) and *CNA1*. (**H**) Wild-type cells were grown at 25 °C until exponential phase and treated with 1 M sodium chloride (NaCl) for the indicated time, and total RNAs were prepared. The mRNA levels were quantified by qRT-PCR analysis, and relative mRNA levels were calculated using *ACT1* mRNA. The data show mean ± SEM (n = 3). NS, not statistically significant (*P* > 0.05), by Tukey’s test. (**I**) Effects of *P*_*CMP2*_*-CMP2* or *P*_*CNA1*_*-CMP2* integrations on salt sensitivity. Wild-type (WT) and *cmp2* mutant strains harboring the integration of *P*_*CMP2*_*-CMP2* or *P*_*CNA1*_*-CMP2* were spotted onto YPD medium lacking or containing 1.0 M sodium chloride (NaCl) and incubated at 25 °C.

The *CMP2* gene encodes a catalytic subunit of calcineurin phosphatase^18,19^. Calcineurin is a heterodimer composed of the catalytic and regulatory subunits^30^. The budding yeast contains another gene encoding a catalytic subunit of calcineurin, which is termed *CNA1*^18,19^. The calcineurin regulatory subunit is encoded by the *CNB1* gene^31^. As shown above, cells lacking Cmp2 exhibited hypersensitivity to ER stress (Fig. 1D). To investigate whether Cna1 is also involved in ER stress response, we examined tunicamycin sensitivity of the *cna1* mutant cells, together with the *cnb1* mutant cells (Fig. 4A). The *cna1* mutation alone did not lead to ER stress hypersensitivity, but significantly enhanced ER stress sensitive phenotype caused by *cmp2* deletion. ER stress hypersensitivity of the *cmp2 cna1* double mutant cells was indistinguishable from that of the *cnb1* mutant cells. These results indicate that Cmp2 and Cna1 act as the major and minor catalytic subunit of calcineurin in ER stress response, respectively.

We next asked why *CMP2* is more important in ER stress response than *CNA1*. We compared the expression levels of Cmp2 and Cna1 using yeast strains carrying the carboxyl-terminally Myc-tagged genes. Consistent with an increase in *CMP2* mRNA levels after ER stress treatment, Cmp2 expression was upregulated by ER stress (Fig. 4B). In contrast, Cna1 expression remained relatively unchanged following exposure to ER stress (Fig. 4B). Under ER stress conditions, Cmp2 was expressed at significantly higher levels than Cna1 (Fig. 4B). Next, we examined whether the difference in the protein levels between Cmp2 and Cna1 is due to the difference in the promoter activity between the *CMP2* and *CNA1* genes. To address this, we generated two reporter constructs, *P*_*CMP2*_*-GFP* and *P*_*CNA1*_*-GFP*, which express *GFP* under the control of the *CMP2* and *CNA1* promoters, respectively (Fig. 4C). *GFP* expression from the *P*_*CMP2*_*-GFP* reporter, but not the *P*_*CNA1*_*-GFP* reporter, was increased after ER stress treatment (Fig. 4D). The *CMP2* promoter was more active than the *CNA1* promoter under ER stress conditions (Fig. 4D). Thus, the difference in the promoter activity between *CMP2* and *CNA1* is reflected in their protein levels.

It may be possible that higher functional significance of Cmp2 attributes to higher activity of the *CMP2* promoter. To explore this possibility, we generated two integrations: one expresses *CMP2* from its own promoter; the other expresses *CMP2* from the *CNA1* promoter. We compared their ability to rescue ER stress hypersensitivity associated with *cmp2* deletion (Fig. 4E). The *P*_*CMP2*_*-CMP2* integration could fully rescue ER stress sensitive phenotype of the *cmp2* mutant. However, the *P*_*CNA1*_*-CMP2* integration failed to restore ER stress hypersensitivity caused by *cmp2* mutation, suggesting that the *CMP2* promoter is critical for the role of the *CMP2* gene in ER stress response. We next examined whether *CNA1* driven by the *CMP2* promoter can compensate for loss of *CMP2*. However, the *cmp2* mutant cells carrying the *P*_*CMP2*_*-CNA1* integration exhibited hypersensitivity to ER stress. This result suggests that not only the promoter but also the sequence including the coding and 3′ untranslated regions are important for the role of the *CMP2* gene in ER stress response.

We next compared the functional significance of Cmp2 and Cna1 in other stress response except for ER stress. Previously, it has been reported that the yeast calcineurin functions in adaptation to high salt conditions^32,33^. Consistent with the previous report^32^, hypersensitivity to sodium ions was observed in the *cmp2* single mutant, but not in the *cna1* single mutant (Fig. 4F). The *cmp2 cna1* double mutant cells exhibited hypersensitivity to sodium ions indistinguishable from that of the *cnb1* mutant cells (Fig. 4F). These results indicate that similar to ER stress response, Cmp2 is more important than Cna1 in salt stress response. However, unlike ER stress response, not only *CNA1* but also *CMP2* mRNA levels were largely unchanged in salt stress response (Fig. 4G,H). We next asked whether the *CMP2* promoter is critical for the role of the *CMP2* gene in salt stress response. The *P*_*CMP2*_*-CMP2* integration completely rescued salt stress sensitive phenotype seen in *cmp2* mutant cells (Fig. 4I). We found that even when *CMP2* was expressed from the *CNA1* promoter, salt stress hypersensitivity associated with *cmp2* mutation could be effectively restored (Fig. 4I). Thus, the *CMP2* promoter is less important for salt stress response compared with ER stress response.

## Discussion

Reversible protein phosphorylation exerted by the antagonistic activity of protein kinases and phosphatases is one of the most well-analyzed post-translational modifications. Numerous studies have demonstrated that proper modulation of phosphorylation is required for eukaryotic cells to adapt to the environmental stress. In controlling the function of protein phosphatases, regulation of their expression levels is frequently utilized. Under ER stress conditions, yeast cells facilitate the expression of a variety of genes functioning to restore ER homeostasis. However, it remains unclear whether the expression levels of protein phosphatases are regulated in yeast ER stress response.

In this study, we examined the mRNA expression patterns of the budding yeast protein phosphatases during ER stress response. Of 35 mRNAs encoding known or putative protein phosphatases, 8 mRNAs (*CMP2*, *MIH1*, *PPZ2*, *PTC6*, *PTP1*, *PTP2*, *SAL6*, and *SDP1*) were upregulated by both chemical and genetic ER stressors; 2 mRNAs (*PTC5* and *YCH1*) were increased by chemical ER stressors, but not by the genetic ER stressor. To investigate the physiological role of ER stress-induced protein phosphatases in ER stress response, we examined ER stress sensitivity associated with each deletion mutant. Contrary to our expectation, only two mutations, *ptp2* and *cmp2*, altered cellular sensitivity to ER stress. In the previous study^34^, double mutants for 30 genes encoding non-essential yeast protein phosphatases were constructed in 435 combinations, and 4 double mutant cells displayed synthetic growth defects under specific conditions. In 10 protein phosphatases that were identified here as those induced by chemical ER stressors, for instance, two mitochondrial protein phosphatases, Ptc5 and Ptc6, are included^35–37^. Additionally, the budding yeast possesses Ptc7, the third protein phosphatase residing within mitochondria^35,36^. Therefore, failure of single mutation to alter ER stress sensitivity may be due to the functional redundancy between more than two protein phosphatases.

Here, we revealed that the Mpk1-Rlm1 axis promotes *PTP2* expression in ER stress response. Previous reports showed that *PTP2* expression is induced by environmental stresses, such as heat shock and osmotic stresses^21,27,28,38^. Upon heat shock, *PTP2* is upregulated in a manner dependent on Mpk1 and Rlm1^27,28^. Thus, it is likely that a similar mechanism operates in regulation of *PTP2* expression in various stress responses. However, the roles of *PTP2* in heat shock and ER stress responses seem to differ from each other: Ptp2 acts to downregulate Mpk1 in heat shock response, whereas Ptp2 functions in Hog1 inactivation during ER stress response^6,27,28^. To date, the physiological necessity of *PTP2* upregulation in heat shock response remains to be elucidated. We showed here that the ER stress resistant phenotype of the *ptp2* mutants was only partially rescued by *PTP2* expression from mutated *PTP2* promoter in which two putative Rlm1-binding motifs were deleted. This result indicates that *PTP2* induction is required for its full function in ER stress response. Taken together, our results clearly demonstrated that the mechanism by which Hog1 is downregulated by the Mpk1-Rlm1 axis through Ptp2 induction exists in the budding yeast ER stress response (Supplementary Fig. 16H). Previous studies have revealed that constitutive activation of Hog1 leads to cell lethality^39,40^. However, under our laboratory conditions used in this study, Hog1 upregulation caused by *ptp2* mutation advantageously functions in adaptation to ER stress, since the *ptp2* mutant cells were resistant to ER stress. Furthermore, *PTP2* overexpression failed to suppress ER stress hypersensitivity caused by *rlm1* mutation, but rather enhanced it (Supplementary Fig. 16G). Nevertheless, why do yeast cells possess the mechanism by which Hog1 is negatively regulated through Ptp2 induction in response to ER stress? Previous studies suggested that ER stress sensitivity of the budding yeast is altered by the extracellular environments, including the composition of the culture medium^8,41^. Furthermore, it is well-known that the activity of Hog1 and Mpk1 are dynamically modulated by various environmental conditions^42,43^. Accordingly, the mechanism of Hog1 downregulation whereby the Mpk1-Rlm1 axis activates *PTP2* expression may be required for yeast cells under certain conditions (for example, the combined stress conditions) to facilitate adaptation to ER stress.

Previously, it has been shown that the budding yeast calcineurin phosphatase confers resistance to ER stress^3,11^. However, it remained unclear how two alternative calcineurin catalytic subunits, Cna1 and Cmp2, operate in ER stress response, because the previous results were mainly based on the experiment using an inhibitor of calcineurin. Here, we found that Cmp2 makes a greater contribution to ER stress response than Cna1. Similar relative contribution was seen in salt stress response^32^ (Fig. 4F). Furthermore, it has been reported that Cmp2 is more important for recovery from the mating factor-induced growth arrest than Cna1^44^. Thus, Cmp2 and Cna1 act as the major and minor catalytic subunits in distinct biological processes, respectively. However, the determinant of the relative functional significance appears to be different between, at least, salt and ER stress responses. Our reporter analysis revealed that the *CNA1* promoter is less active than the *CMP2* promoter. Nevertheless, Cmp2 expression from the *CNA1* promoter could almost completely rescue the salt sensitive phenotype associated with *cmp2* mutation. This result suggests that higher promoter activity is not required for the *CMP2* gene to confer salt tolerance. In contrast, the promoter activity is likely to be a key determinant of the functional significance in ER stress response, since replacement of the *CMP2* promoter with the *CNA1* promoter considerably reduced the function of *CMP2* in ER stress response. Additionally, the *CMP2* gene may possess another element that is functionally important for ER stress response. This idea is derived from our observation that Cna1 expression from the *CMP2* promoter hardly rescued *cmp2* hypersensitivity to ER stress. Our results presented here suggest that the adaptive response to ER stress requires a higher amount of Cmp2 than salt stress. One plausible explanation for this phenomenon is as follows: the Cmp2 targets are different between ER and salt stress responses; more abundant Cmp2 is required for interacting physically with its target in ER stress response. In the budding yeast, a major target of the calcineurin is Crz1 transcription factor^45,46^. Crz1 acts downstream of the calcineurin in salt stress response, whereas Crz1 is unlikely to transduce a signal from the calcineurin in ER stress response^41,45–47^. The previous study suggests that under ER-stressed conditions, the calcineurin dephosphorylates Cch1, a subunit of the high affinity calcium channel^3^. However, it remains obscure whether Cch1 dephosphorylation is related to the physiological role of the calcineurin in ER stress response. Thus, identification of the calcineurin target in ER stress response should be needed to elucidate why higher promoter activity is required for the function of *CMP2* in ER stress response. Additionally, since *CMP2* induction occurred in the *hac1*, *rlm1*, *hog1* and *snf1* mutant cells (Mizuno *et al*., unpublished data), identification of the components involved in *CMP2* expression should be needed for the understanding of the regulatory mechanisms of the calcineurin in ER stress response.

## Materials and Methods

### Plasmids

The *PTP2*, *CMP2* and *CNA1* genes were amplified from the *Saccharomyces cerevisiae* W303 derivative^48^ by PCR with the following primers: 5′-CTCTAGAGGATCCCCGGGGGACACTCGTTTAATTTAGC-3′ and 5′-TCGAGCTCGGTACCCGGGTATGGGTACTGACATCTCTG-3′ for *PTP2*; 5′-CTCTAGAGGATCCCCGGGCGTCCCAAAAAGGAAATAGC-3′ and 5′-TCGAGCTCGGTACCCGGGTCTCTGAGTCAGACAGTGTC-3′ for *CMP2*; 5′-CTCTAGAGGATCCCCGGGGATTTTGAAGATACTAGTGC-3′ and 5′- TCGAGCTCGGTACCCGGGTTGGTCGCACAAGGTGTCTC-3′ for *CNA1*. The amplified *PTP2*, *CMP2* and *CNA1* DNA fragments were inserted into the YCplac33 vector^49^ by In-Fusion cloning kits (Takara), yielding the YCplac33-P_PTP2_-PTP2, YCplac33-P_CMP2_*-*CMP2 and YCplac33-P_CNA1_*-*CNA1 plasmids, respectively. To make the *P*_*PTP2*_*-GFP*, *P*_*CMP2*_*-GFP* and *P*_*CNA1*_*-GFP* constructs, 1000-bp, 1000-bp and 997-bp genomic fragments containing the 5′ upstream sequences of the *PTP2*, *CMP2* and *CNA1* genes, respectively, were amplified by PCR with the following primers: 5′-CTCTAGAGGATCCCCGGGGGACACTCGTTTAATTTAGC-3′ and 5′-TAACCCGGGGATCCGATCCATCAATAGCAACGTCGATC-3′ for *P*_*PTP2*_*-GFP*; 5′-CTCTAGAGGATCCCCGGGCGTCCCAAAAAGGAAATAGC-3′ and 5′-TAACCCGGGGATCCGAGACATTGCGGGTTCAAGAAG-3′ for *P*_*CMP2*_*-GFP*; 5′-CTCTAGAGGATCCCCGGGGATTTTGAAGATACTAGTGC-3′ and 5′-TAACCCGGGGATCCGCGACATTGGCGTTGAGAGTG-3′ for *P*_*CNA1*_*-GFP*. The DNA fragment encoding GFP followed by the *ADH1* terminator (*T*_*ADH1*_) was amplified from the pFA6a-GFP-HIS3MX6 vector by PCR with the following primers: 5′-CGGATCCCCGGGTTAATTAAC-3′ and 5′-TCGAGCTCGGTACCCGGGAGATCTATATTACCCTGTTATCC-3′. The amplified 5′ upstream sequences of the *PTP2*, *CMP2* and *CNA1* genes, together with the *GFP-T*_*ADH1*_ DNA fragment, were fused to the YCplac33 vector by In-Fusion cloning kits (Takara), yielding the YCplac33-P_PTP2_-GFP, YCplac33-P_CMP2_-GFP and YCplac33-P_CNA1_-GFP plasmids, respectively. Deletions of RBMs in the *PTP2* promoter were generated by oligonucleotide-directed PCR using the YCplac33-P_PTP2_-GFP plasmid as a template. The primers used to delete RBMs are: 5′-CTACACATAAAGTTCCATAAAGCAG-3′ and 5′-GAACTTTATGTGTAGTACACCTAAC-3′ for RBM1 deletion; 5′-TGTAAACACTGGGGATCGGACCTAG-3′ and 5′-TCCCCAGTGTTTACAATAAAATAGG-3′ for RBM2 deletion. The *P*_*ACT1*_*-PTP2* construct was generated as follows. A 728-bp genomic fragment containing the *ACT1* promoter was amplified from the *Saccharomyces cerevisiae* W303 derivative by PCR with the following primers: 5′- CTCTAGAGGATCCCCGGGAAGGGAACGTCAACCTGAAG-3′ and 5′-TGCTATGCGATCCATTGTTAATTCAGTAAATTTTCG-3′. The coding region of the *PTP2* gene together with the 3′ downstream sequence was amplified by PCR with the following primers: 5′- ATGGATCGCATAGCACAGCAATATCG-3′ and 5′-TCGAGCTCGGTACCCGGGTATGGGTACTGACATCTCTG-3′. The amplified P_ACT1_ and *PTP2* DNA fragments were fused with the YCplac33 vector by In-Fusion cloning kits (Takara), yielding the YCplac33-P_ACT1_-PTP2 plasmid. The *P*_*CNA1*_*-CMP2* construct was generated as follows. The *CNA1* promoter was amplified by PCR with the following primers: 5′-CTCTAGAGGATCCCCGGGGATTTTGAAGATACTAGTGC-3′ and 5′-AGCGTCTGAAGACATTGGCGTTGAGAGTGTTTTATGG-3′. The coding region of the *CMP2* gene together with the 3′ downstream sequence was amplified by PCR with the following primers: 5′-ATGTCTTCAGACGCTATAAGAAATAC-3′ and 5′-TCGAGCTCGGTACCCGGGTCTCTGAGTCAGACAGTGTC-3′. The amplified P_*CNA1*_ and *CMP2* DNA fragments were fused with the YCplac33 vector by In-Fusion cloning kits (Takara), yielding the YCplac33-P_CNA1_-CMP2 plasmid. Similarly, the YCplac33-P_CMP2_-CNA1 plasmid was constructed. The primers used to amplify P_*CMP2*_ and *CNA1* are: 5′-CTCTAGAGGATCCCCGGGCGTCCCAAAAAGGAAATAGC-3′ and 5′-CAAGTCTTTCGACATTGCGGGTTCAAGAAGAAG-3′ for *P*_*CMP2*_; 5′-ATGTCGAAAGACTTGAATTCTTCACG-3′ and 5′-TCGAGCTCGGTACCCGGGTTGGTCGCACAAGGTGTCTC-3′ for *CNA1*. To generate the integrations, the inserts in the YCplac33 plasmids were subcloned into the pRS306 vector^50^. Plasmids used in this study are described in Supplementary Table 1.

### Strains

Standard procedures were followed for yeast manipulations^51^. Yeast strains harboring the complete gene deletions (*MIH1*, *SDP1*, *YCH1*, *PTC5*, *PTC6*, *CMP2*, *PPZ2*, *SAL6*, *PTP1*, *MPK1*, *RLM1*, *CNA1* and *CNB1*) and carboxyl-terminally Myc-tagged genes (*CMP2* and *CNA1*) were generated by a PCR-based method as described previously^52,53^. Primer sets were designed such that 46 bases at the 5′ end of primers were complementary to those at the corresponding region of the target gene, and 20 bases at their 3′ end were complementary to the pFA6a sequence, 5′-TGCAGTACTCTGCGGGTGTATACAG-3′ or 5′- ATTTGACTGTATTACCAATGTCAGC-3′. All strains produced by a PCR-based method were verified by colony PCR amplification to confirm that replacement had occurred at the expected locus. Strains carrying the integrations, *P*_*PTP2*_*-GFP*, *P*_*CMP2*_*-GFP*, *P*_*CNA1*_*-GFP*, *P*_*PTP2*_*-PTP2*, *P*_*ACT1*_*-PTP2*, *P*_*CMP2*_*-CMP2*, *P*_*CNA1*_*-CNA1*, *P*_*CNA1*_*-CMP2*, and *P*_*CMP2*_*-CNA1*, were constructed by integrating the linearized plasmids, pRS306-P_PTP2_-GFP, pRS306-P_CMP2_-GFP, pRS306-P_CNA1_-GFP, pRS306-P_PTP2_-PTP2, pRS306-P_ACT1_-PTP2, pRS306-P_CMP2_-CMP2, pRS306-P_CNA1_-CNA1, pRS306-P_CNA1_-CMP2, and pRS306-P_CMP2_-CNA1, respectively. The *sec12-4*, *sec13-1* and *sec16-2* strains of the YPH499 derivative were kind gifts from Dr. Akihiko Nakano (Riken). Strains used in this study are listed in Supplementary Table 2.

### RNA isolation and RT–PCR

Preparation of total RNA and generation of cDNA were performed as described previously^9^. The cDNAs were quantitated by a quantitative real-time RT-PCR (qRT-PCR) method using a 7500 fast real-time RT-PCR system (Applied Biosystems) with SYBR Premix Ex Taq (Takara), and levels of gene expression were normalized to *ACT1* expression. Primers used to analyze the mRNA level are described in Supplementary Table 3.

### Protein extraction, western blot analysis and antibodies

Preparation of protein extracts and Western blot analysis were performed as described previously^9^. Anti-phospho-p38 MAPK monoclonal antibody D3F9 (Cell Signaling), anti-Hog1 polyclonal antibody y-215 (Santa Cruz), anti-phospho-p44/42 MAPK polyclonal antibody (Cell Signaling), anti-Mpk1 polyclonal antibody yN-19 (Santa Cruz), anti-Myc monoclonal antibody 9E10 (Santa Cruz) and anti-Mcm2 polyclonal antibody N-19 (Santa Cruz) were used. Detection was carried out by using a LAS-4000 (Fuji Film) with Immobilon Western (Merck Millipore) or the Odyssey Imaging Systems (LI-COR Biosciences). Signal intensities were quantified by the Odyssey Imaging Systems, and statistical analysis was performed with Excel (Microsoft).

### Stress sensitivity

Assays for tunicamycin and sodium chloride toxicity were carried out as follows. Cells were grown to exponential phase, and cultures were adjusted to an optical density of 0.5. Cell cultures were then serially diluted 5-fold, spotted onto normal plates or plates containing the indicated concentrations of tunicamycin, followed by incubation at 25 °C for 3 days (for plates lacking stressors) or more than 5 days (for plates containing stressors).

## Electronic supplementary material

Supplemental information
