# Supplementary material for: Induction of Ptp2 and Cmp2 protein phosphatases is crucial for the adaptive response to ER stress in Saccharomyces cerevisiae
Source: Sci Rep. 2018 Aug 30;8:13078. doi: 10.1038/s41598-018-31413-6 (PMC6117328; doi:10.1038/s41598-018-31413-6)
Supplement: Supplementary file 1 — Supplemental information [file 41598_2018_31413_MOESM1_ESM.pdf]

Title:

Induction of Ptp2 and Cmp2 protein phosphatases is crucial for the adaptive response to ER stress in *Saccharomyces cerevisiae*

Authors:

Tomoaki Mizuno*, Meyu Nakamura and Kenji Irie

Affiliation:

Department of Molecular Cell Biology, Faculty of Medicine, University of Tsukuba, Tsukuba, Japan.

*Correspondence and requests for materials should be addressed to T. M. (mizuno@md.tsukuba.ac.jp)

Supplementary Figure 1

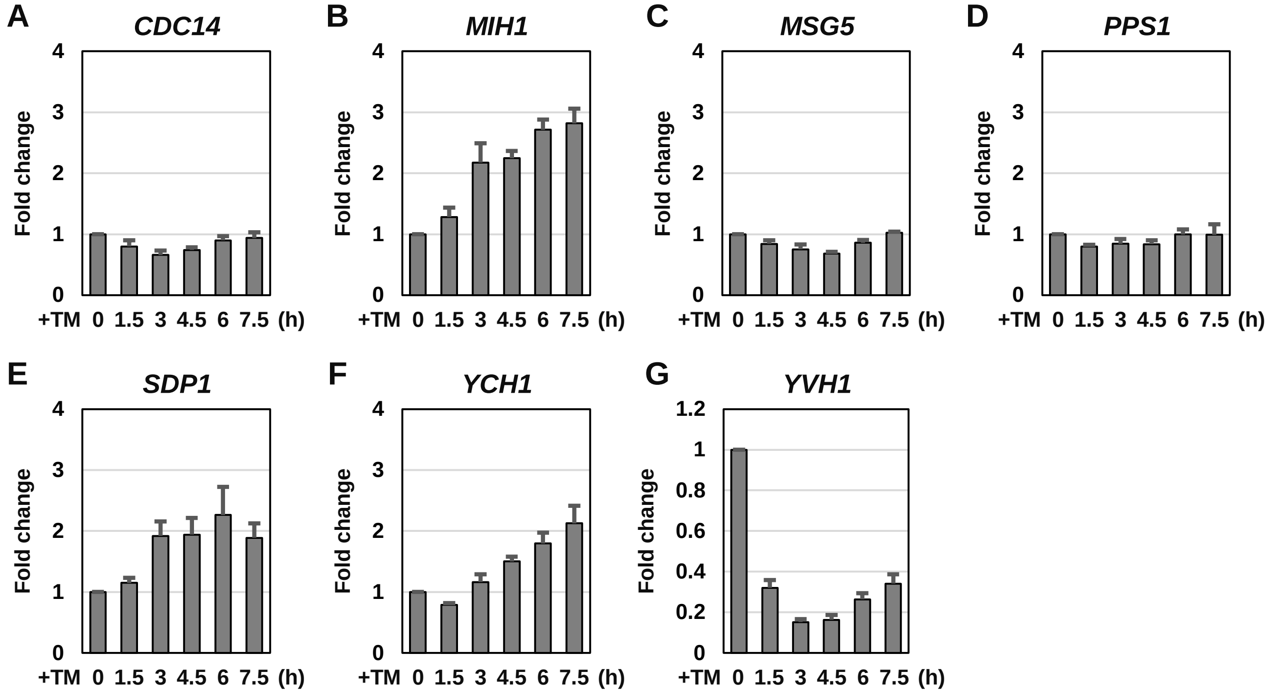

Supplementary Figure 2

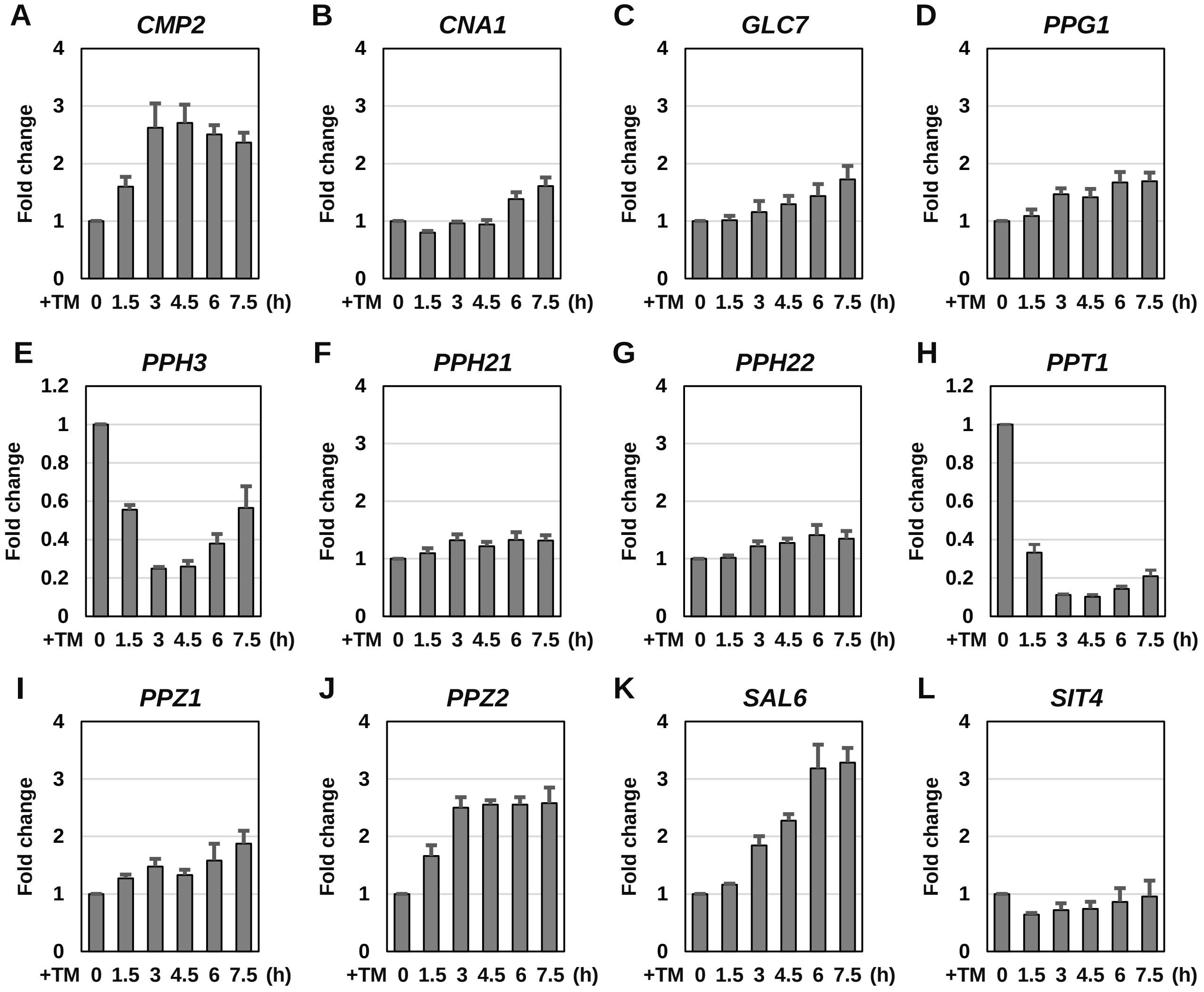

Supplementary Figure 3

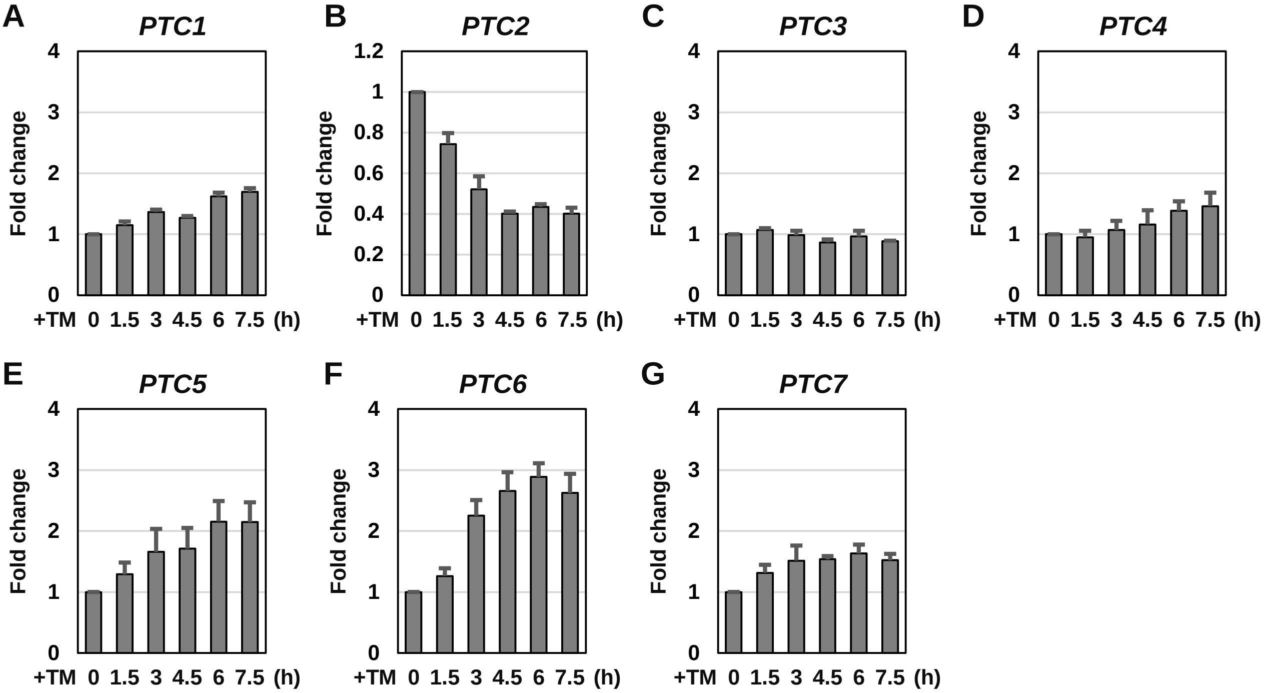

Supplementary Figure 4

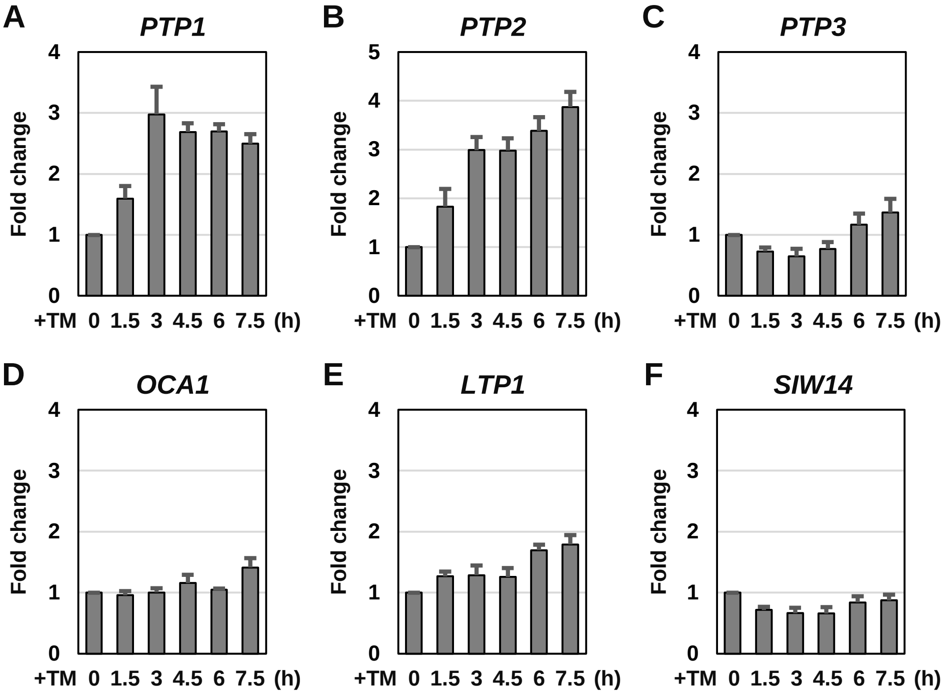

Supplementary Figure 5

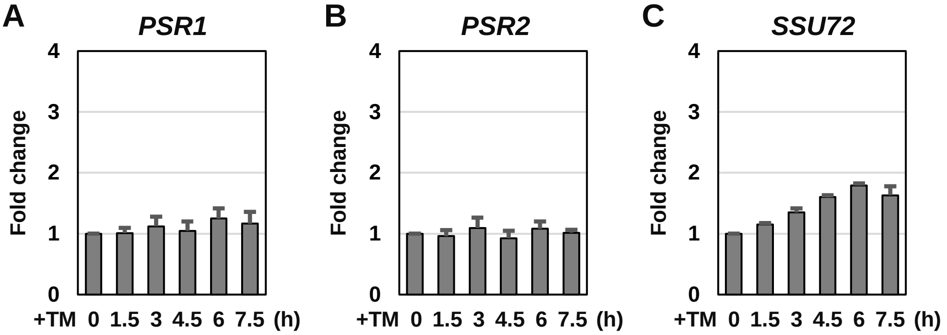

Supplementary Figure 6

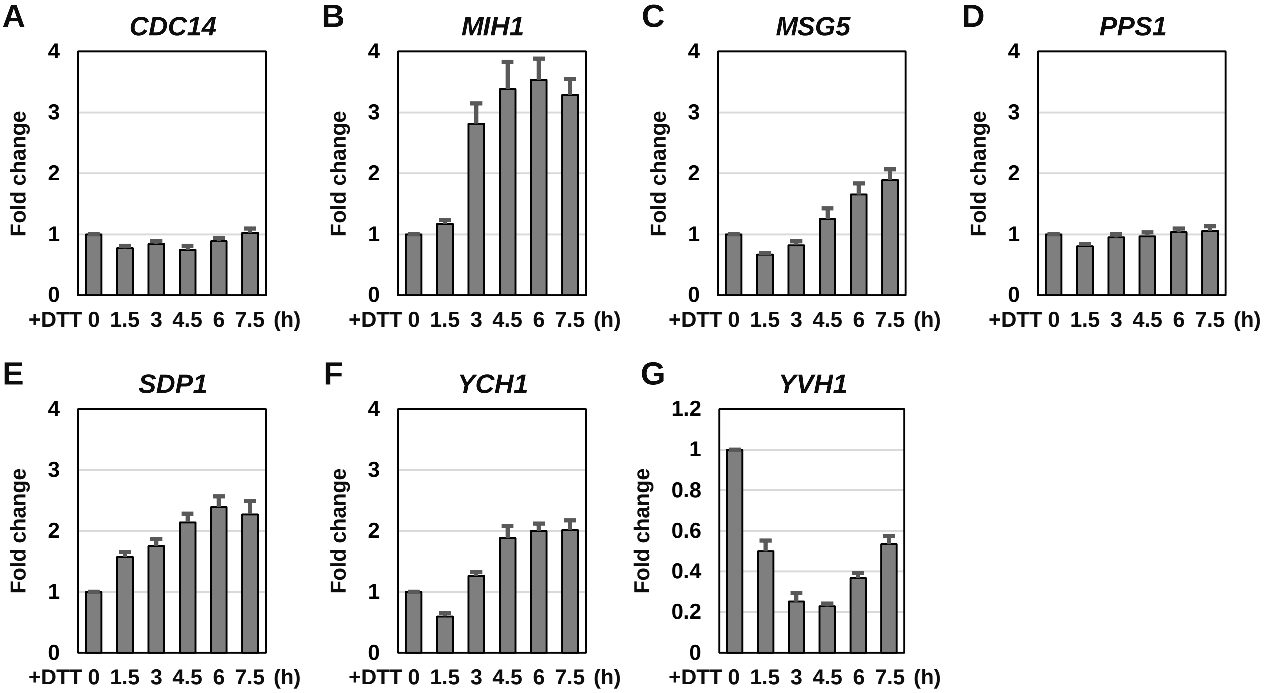

Supplementary Figure 7

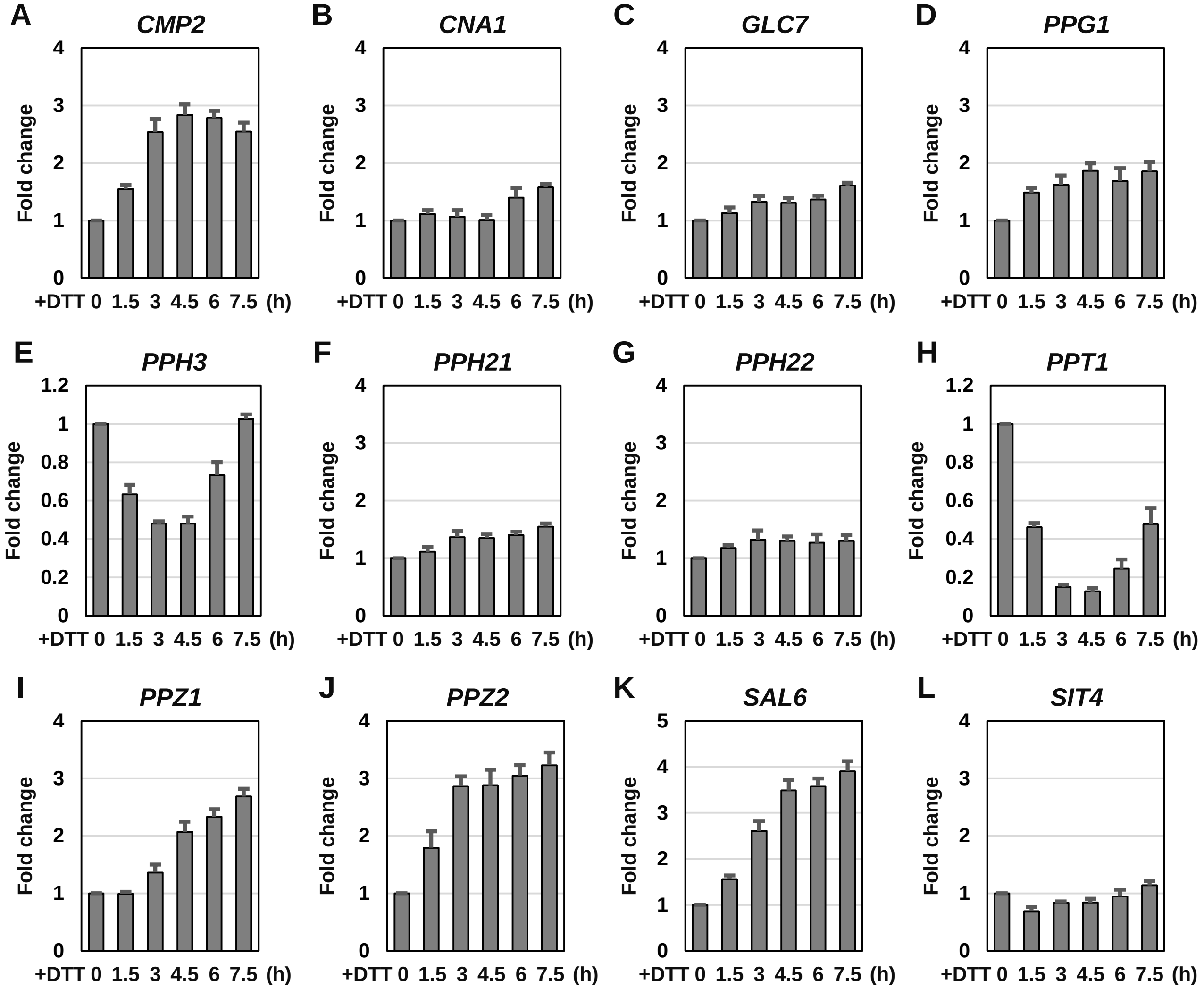

Supplementary Figure 8

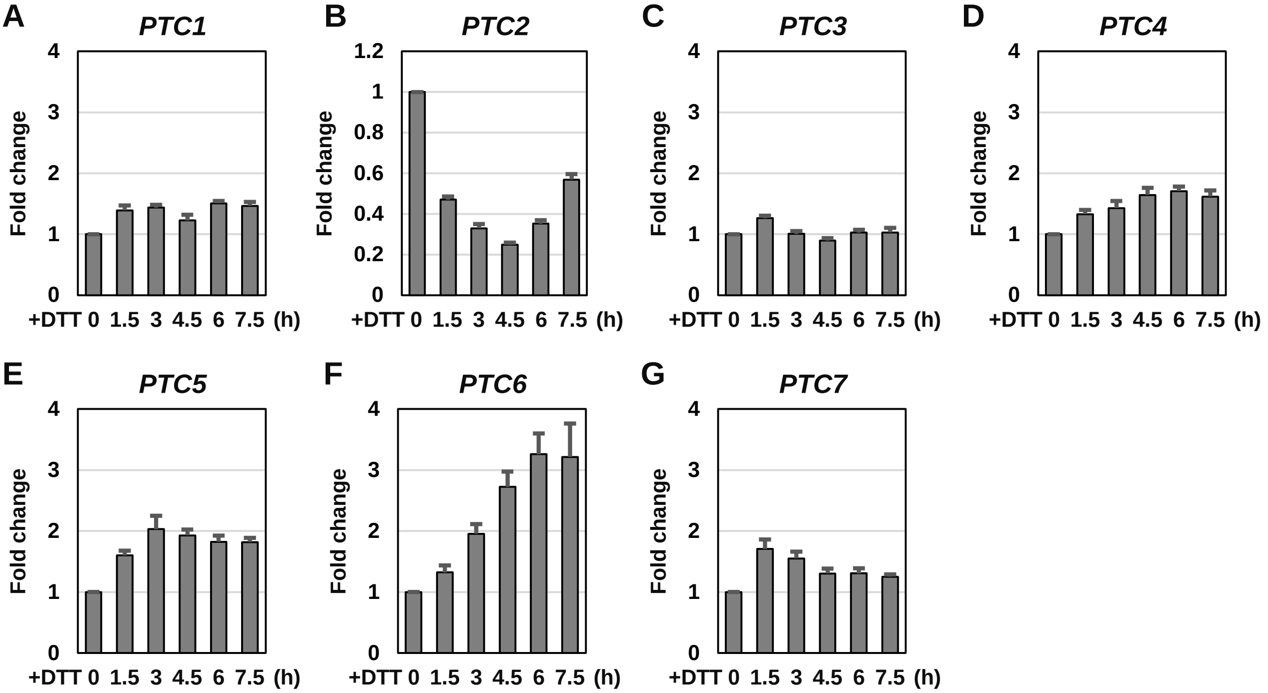

Supplementary Figure 9

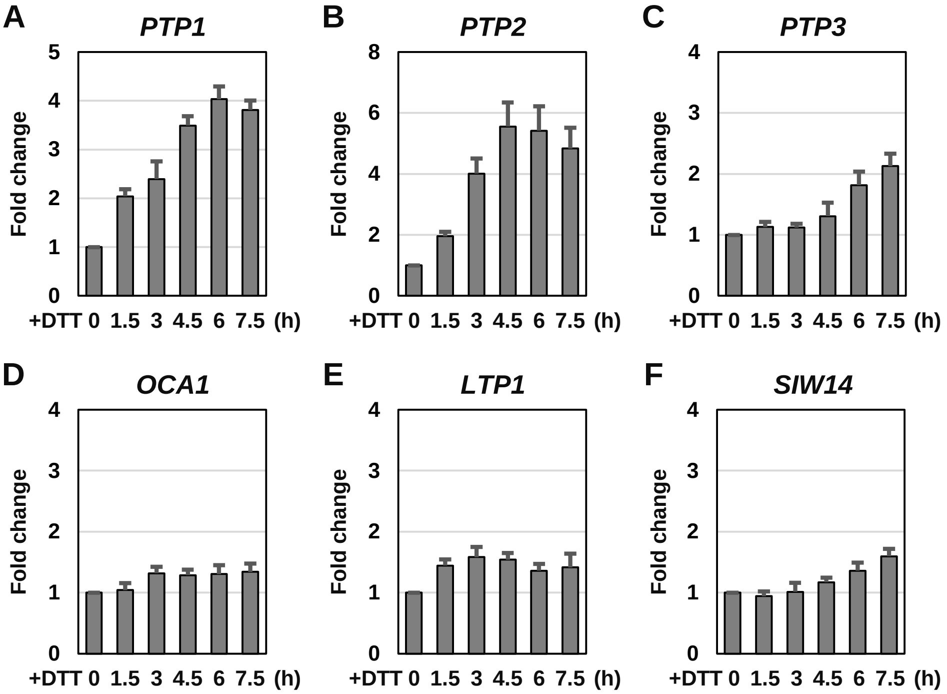

Supplementary Figure 10

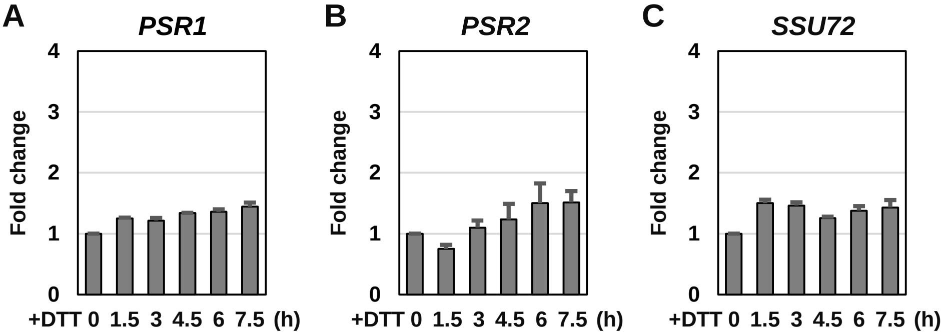

Supplementary Figs. 1-10. The mRNA levels of 35 genes encoding protein phosphatases during ER stress response.

Wild-type (WT) cells were grown at 25 ^{o}C until exponential phase and treated with 2 µg/ml tunicamycin (TM) (Supplementary Figs. 1-5) and 4 mM dithiothreitol (DTT) (Supplementary Figs. 6-10) for the indicated time, and total RNAs were prepared. The mRNA levels were quantified by qRT-PCR analysis, and relative mRNA levels were calculated using *ACT1* mRNA. (Supplementary Figs. 1, 6) 7 genes that encode protein phosphatases belonging to the dual specificity phosphatase (DSP) family. (Supplementary Figs. 2, 7) 12 genes that encode protein phosphatases belonging to the phosphoprotein phosphatase (PPP) family. (Supplementary Figs. 3, 8) 7 genes that encode protein phosphatases belonging to the protein phosphatase Mg^{2+}- or Mn^{2+}- dependent (PPM) family. (Supplementary Figs. 4, 9) 6 genes that encode protein phosphatases belonging to the protein tyrosine phosphatase (PTP) family. (Supplementary Figs. 5, 10) 3 genes that encode ungrouped protein phosphatases. The data show mean ± SEM (n = 3).

Supplementary Figure 11

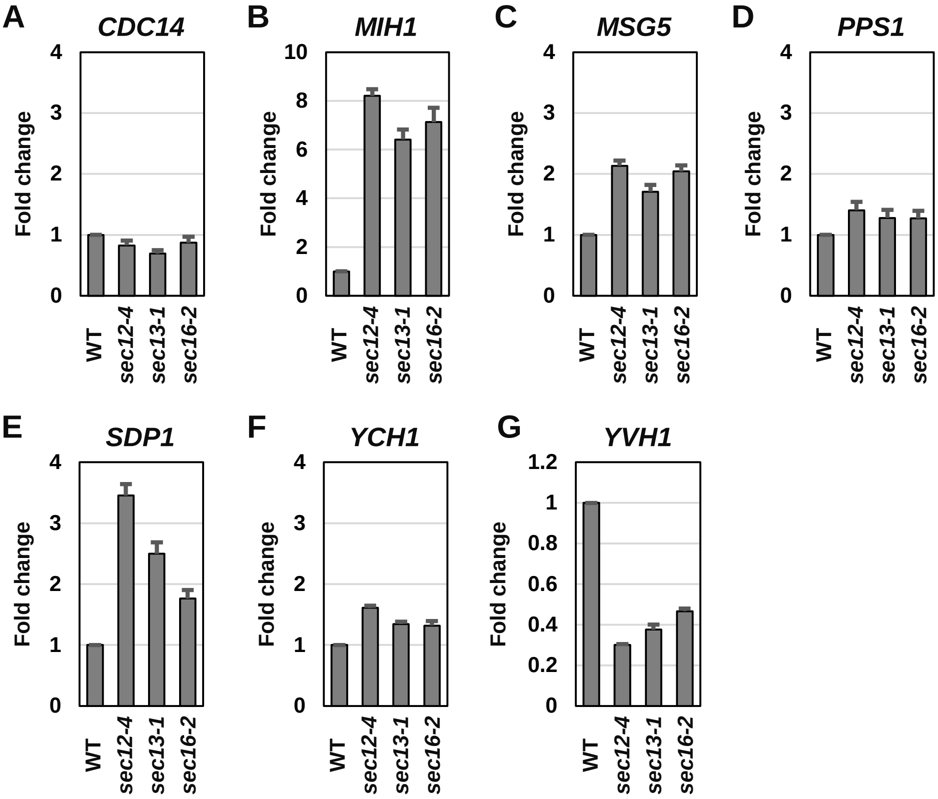

Supplementary Figure 12

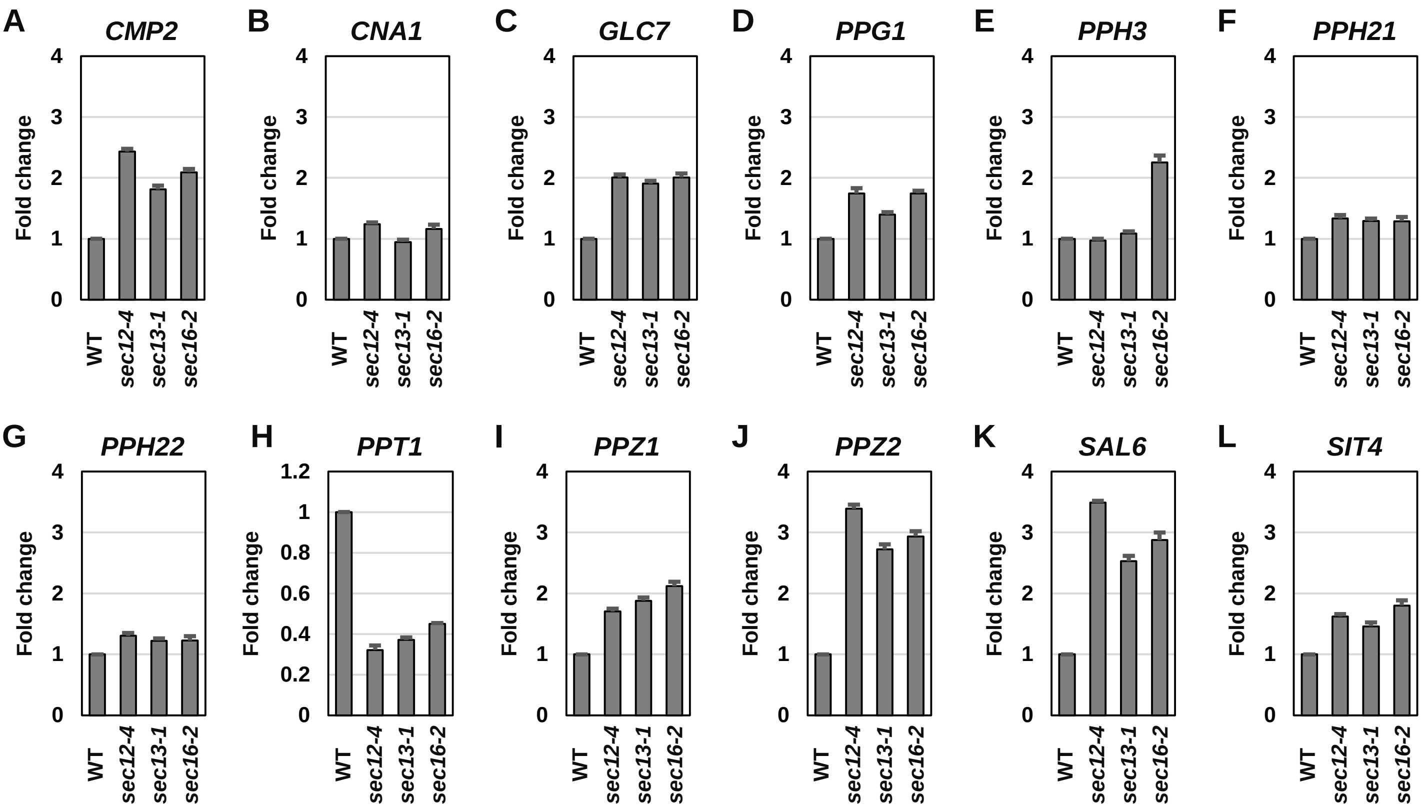

Supplementary Figure 13

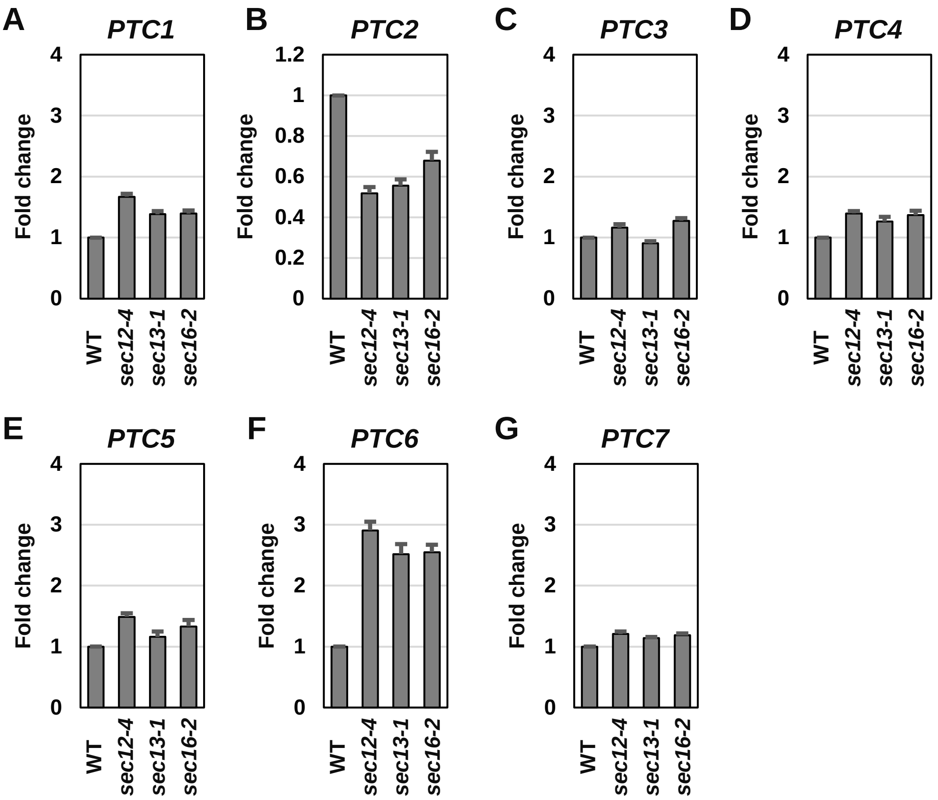

Supplementary Figure 14

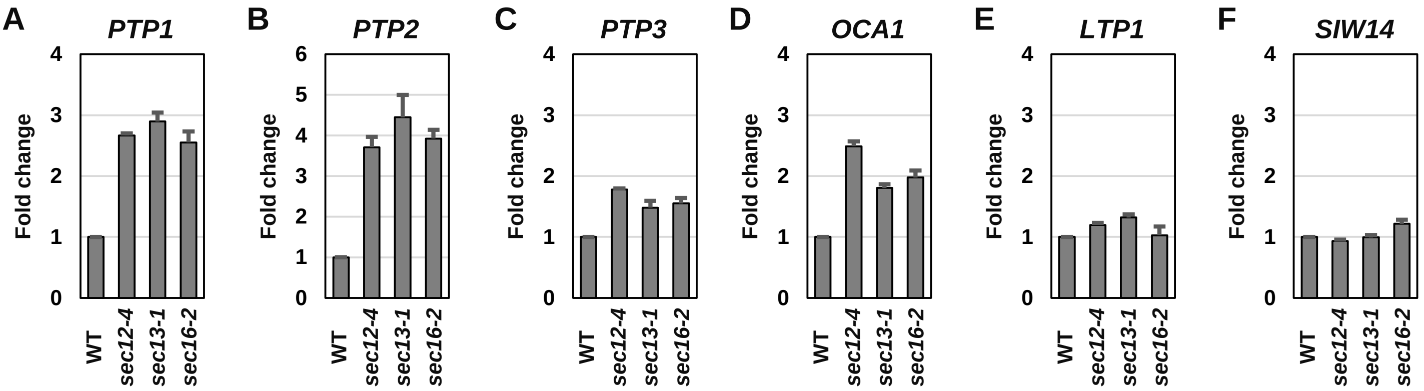

Supplementary Figure 15

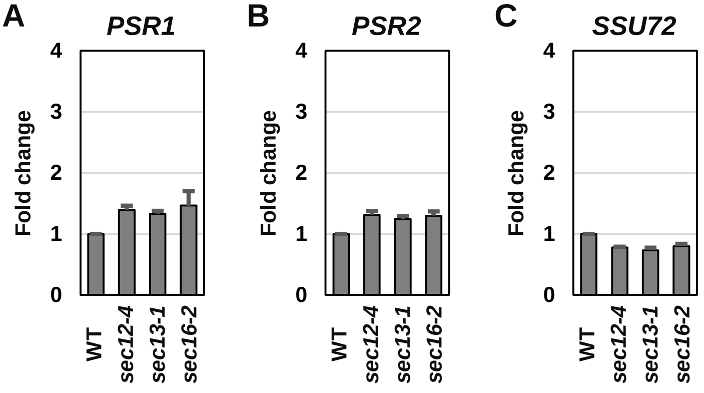

Supplementary Figs. 11-15. The mRNA levels of 35 genes encoding protein phosphatases in *sec* mutant cells.

Wild-type (WT) and *sec12-4*, *sec13-1* and *sec16-2* mutant cells were grown at 25 ^oC until exponential phase and incubated at 37 ^oC for 4 hours, and total RNAs were prepared. The mRNA levels were quantified by qRT-PCR analysis, and relative mRNA levels were calculated using *ACT1* mRNA. (Supplementary Figs. 11) 7 genes that encode protein phosphatases belonging to the dual specificity phosphatase (DSP) family. (Supplementary Figs. 12) 12 genes that encode protein phosphatases belonging to the phosphoprotein phosphatase (PPP) family. (Supplementary Figs. 13) 7 genes that encode protein phosphatases belonging to the protein phosphatase Mg^{2+}- or Mn^{2+}- dependent (PPM) family. (Supplementary Figs. 14) 6 genes that encode protein phosphatases belonging to the protein tyrosine phosphatase (PTP) family. (Supplementary Figs. 15) 3 genes that encode ungrouped protein phosphatases. The data show mean ± SEM (n = 3).

Supplementary Figure 16

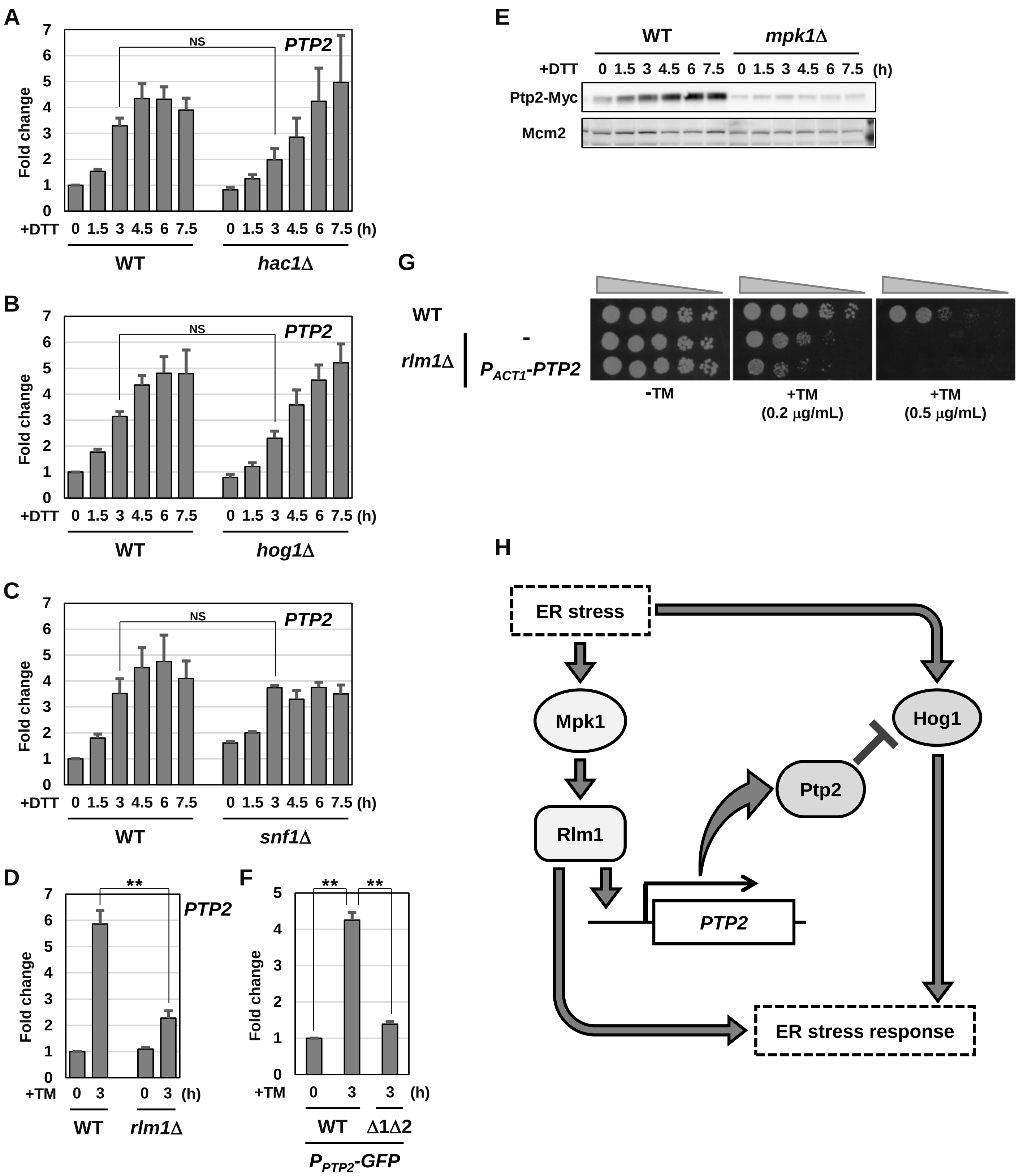

Supplementary Fig. 16. Ptp2 is induced by the Mpk1-Rlm1 axis.
(A-D) The mRNA levels of *PTP2*. Wild-type (WT) and indicated mutant strains were grown at 25 ^{o}C until exponential phase and treated with 4 mM dithiothreitol (DTT) (A-C) or 2 μg/ml tunicamycin (TM) (D) for the indicated time, and total RNAs were prepared from each strain. The mRNA levels were quantified by qRT-PCR analysis, and relative mRNA levels were calculated using *ACT1* mRNA. The data show mean ± SEM. A, B and D, (n = 4); C, (n = 3). $^{**}P < 0.01$ as determined by Tukey's test. NS, not statistically significant ($P > 0.05$).
(E) The protein levels of Ptp2. Wild-type (WT) and *mpk1* mutant strains harboring Myc-tagged *PTP2* were grown at 25 ^{o}C until exponential phase and treated with 4 mM dithiothreitol (DTT) for the indicated time. Extracts prepared from each cell were immunoblotted with anti-Myc and anti-Mcm2 antibodies. Original blots are presented in Supplementary Fig. 23.
(F) Effects of deletion mutations in putative Rlm1-binding motifs on expression of the P_{PTP2}-*GFP* reporter. Wild-type (WT) cells harboring the integration which expresses *GFP* under the control of wild-type or mutated *PTP2* promoter were grown at 25 ^{o}C until exponential phase and treated with 2 μg/ml tunicamycin (TM) for the indicated time. The *GFP* mRNA levels were quantified by qRT-PCR analysis, and relative mRNA levels were calculated using *ACT1* mRNA. The data show mean ± SEM (n = 4). $^{**}P < 0.01$ as determined by Tukey's test.
(G) Effects of the P_{ACT1}-*PTP2* integration on ER stress sensitivity. Wild-type (WT) and *rlm1* mutant strains harboring the P_{ACT1}-*PTP2* integration were spotted onto YPD medium lacking, or containing 0.2 or 0.5 μg/ml tunicamycin (TM) and incubated at 25 ^{o}C for 2 days.
(H) Proposed model for Hog1 downregulation whereby the Mpk1-Rlm1 signaling pathway mediates transcriptional activation of the *PTP2* gene.

Supplementary Figure 17
(Original data for Fig. 2B)

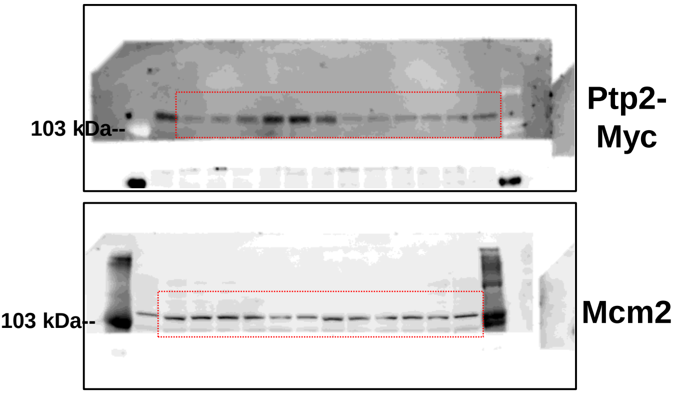

Supplementary Figure 18
(Original data for Fig. 2C)

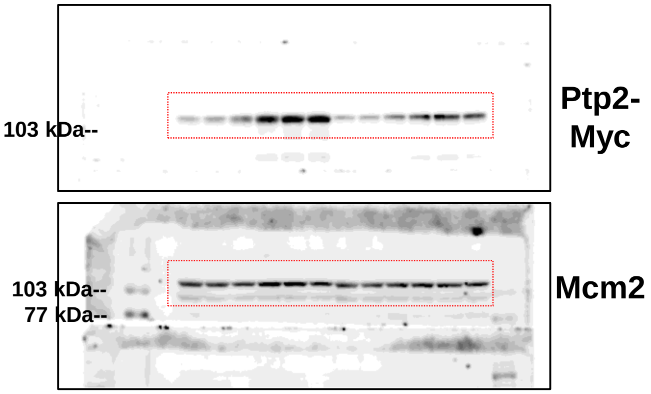

Supplementary Figure 19
(Original data for Fig. 3B)

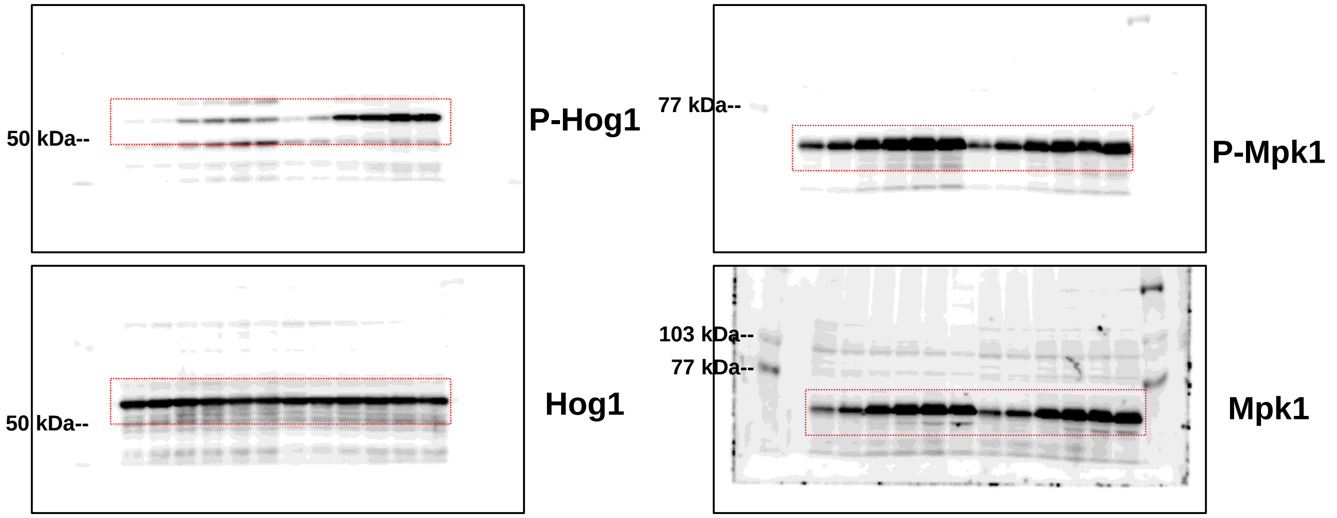

Supplementary Figure 20
(Original data for Fig. 3C)

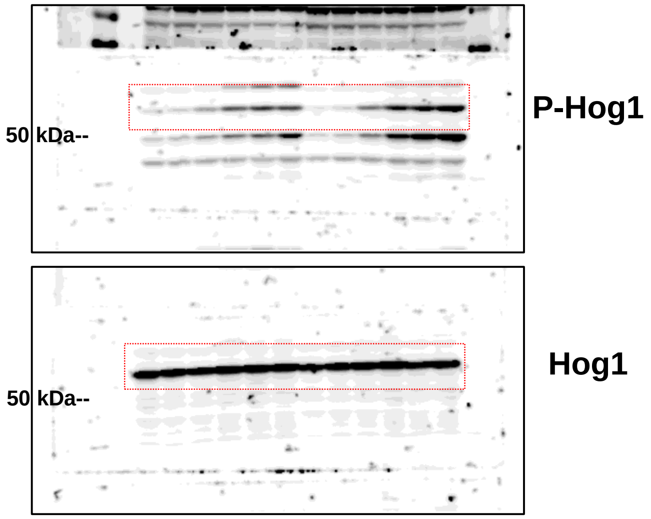

Supplementary Figure 21
(Original data for Fig. 3D)

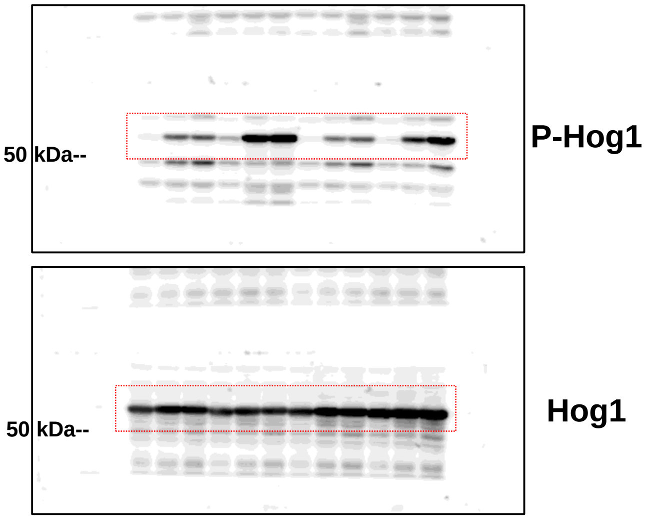

Supplementary Figure 22
(Original data for Fig. 4B)

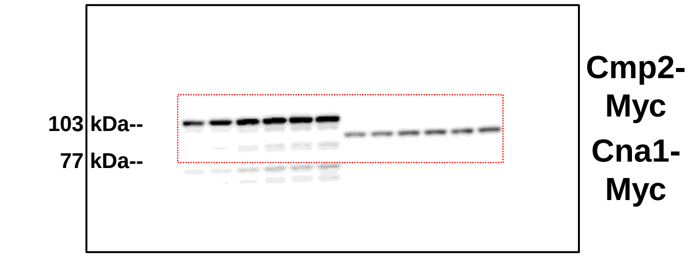

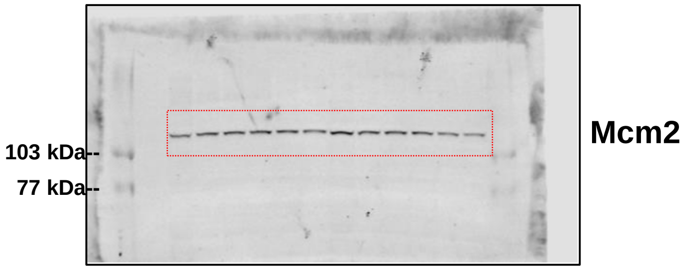

Supplementary Figure 23
(Original data for Supplementary Fig. 16E)

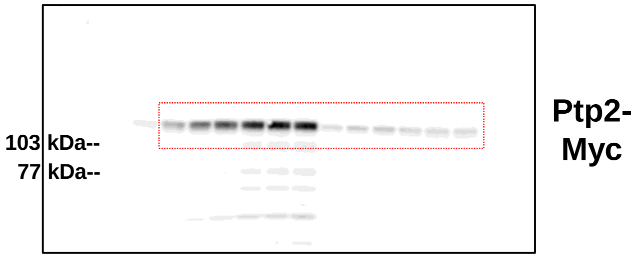

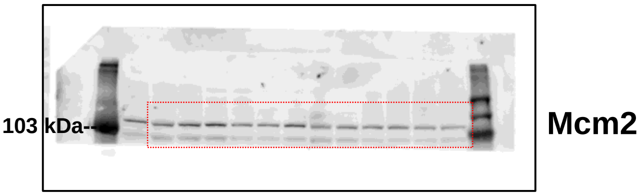

Supplementary Table 1

Plasmids	Relevant markers	Source
pFA6a-kanMX6	*kanMX6*	52
pFA6a–natNT2	*natNT2*	53
pFA6a-GFP-HIS3MX6	*GFP-ADH 3'UTR-HIS3MX6*	52
pFA6a-13Myc-HIS3MX6	*13XMyc-ADH 3'UTR-HIS3MX6*	52
YCplac33	*URA3*	49
pRS306	*URA3*	50
YCplac33-P$_{PTP2}$-GFP	*URA3, P* $_{PTP2}$*-GFP*	this study
YCplac33-P$_{PTP2\Delta1}$-GFP	*URA3, P* $_{PTP2\,\Delta1}$*-GFP*	this study
YCplac33-P$_{PTP2\Delta2}$-GFP	*URA3, P* $_{PTP2\,\Delta2}$*-GFP*	this study
YCplac33-P$_{PTP2\Delta1\Delta1}$-GFP	*URA3, P* $_{PTP2\,\Delta1\,\Delta2}$*-GFP*	this study
YCplac33-P$_{PTP2}$-PTP2	*URA3, P* $_{PTP2}$*-PTP2*	this study
YCplac33-P$_{PTP2\Delta1}$-PTP2	*URA3, P* $_{PTP2\,\Delta1}$*-PTP2*	this study
YCplac33-P$_{PTP2\Delta2}$-PTP2	*URA3, P* $_{PTP2\,\Delta2}$*-PTP2*	this study
YCplac33-P$_{PTP2\Delta1\Delta1}$-PTP2	*URA3, P* $_{PTP2\,\Delta1\,\Delta2}$*-PTP2*	this study
YCplac33-P$_{ACT1}$-PTP2	*URA3, P* $_{ACT1}$*-PTP2*	this study
YCplac33-P$_{CMP2}$-GFP	*URA3, P* $_{CMP2}$*-GFP*	this study
YCplac33-P$_{CNA1}$-GFP	*URA3, P* $_{CNA1}$*-GFP*	this study
YCplac33-P$_{CMP2}$-CMP2	*URA3, P* $_{CMP2}$*-CMP2*	this study
YCplac33-P$_{CNA1}$-CMP2	*URA3, P* $_{CNA1}$*-CMP2*	this study
YCplac33-P$_{CNA1}$-CNA1	*URA3, P* $_{CNA1}$*-CNA1*	this study
YCplac33-P$_{CMP2}$-CNA1	*URA3, P* $_{CMP2}$*-CNA1*	this study
pRS306-P$_{PTP2}$-GFP	*URA3, P* $_{PTP2}$*-GFP*	this study
pRS306-P$_{PTP2\Delta1}$-GFP	*URA3, P* $_{PTP2\,\Delta1}$*-GFP*	this study
pRS306-P$_{PTP2\Delta2}$-GFP	*URA3, P* $_{PTP2\,\Delta2}$*-GFP*	this study
pRS306-P$_{PTP2\Delta1\Delta1}$-GFP	*URA3, P* $_{PTP2\,\Delta1\,\Delta2}$*-GFP*	this study
pRS306-P$_{PTP2}$-PTP2	*URA3, P* $_{PTP2}$*-PTP2*	this study
pRS306-P$_{PTP2\Delta1}$-PTP2	*URA3, P* $_{PTP2\,\Delta1}$*-PTP2*	this study
pRS306-P$_{PTP2\Delta2}$-PTP2	*URA3, P* $_{PTP2\,\Delta2}$*-PTP2*	this study
pRS306-P$_{PTP2\Delta1\Delta1}$-PTP2	*URA3, P* $_{PTP2\,\Delta1\,\Delta2}$*-PTP2*	this study
PRS306-P$_{ACT1}$-PTP2	*URA3, P* $_{ACT1}$*-PTP2*	this study
pRS306-P$_{CMP2}$-GFP	*URA3, P* $_{CMP2}$*-GFP*	this study
pRS306-P$_{CNA1}$-GFP	*URA3, P* $_{CNA1}$*-GFP*	this study
pRS306-P$_{CMP2}$-CMP2	*URA3, P* $_{CMP2}$*-CMP2*	this study
pRS306-P$_{CNA1}$-CMP2	*URA3, P* $_{CNA1}$*-CMP2*	this study
pRS306-P$_{CNA1}$-CNA1	*URA3, P* $_{CNA1}$*-CNA1*	this study
pRS306-P$_{CMP2}$-CNA1	*URA3, P* $_{CMP2}$*-CNA1*	this study

Supplementary Table 2

Strains	Genotype	Source or reference
10B	MAT α ade2 trp1 can1 leu2 his3 ura3 GAL psi+ HOp-ADE2-HO 3' UTR	48
10Ba	MAT **a** ade2 trp1 can1 leu2 his3 ura3 GAL psi+ HOp-ADE2-HO 3' UTR	48
10BD	MAT **a**/MAT α ade2/ade2 trp1/trp1 can1/can1 leu2/leu2 his3/his3 ura3/ura3	48
YCH101	ade2 trp1 can1 leu2 his3 ura3 snf1 Δ::CgTRP1	9
YCH137	ade2 trp1 can1 leu2 his3 ura3 hog1 Δ::kanMX6	9
YCH144	ade2 trp1 can1 leu2 his3 ura3 ptp2 Δ::kanMX6	9
YCH217	ade2 trp1 can1 leu2 his3 ura3 PTP2-Myc::HIS3MX6	9
YCH241	ade2 trp1 can1 leu2 his3 ura3 hac1 Δ::kanMX6	9
YCH133	ade2 trp1 can1 leu2 his3 ura3 mpk1 Δ::kanMX6	this study
YCH134	ade2 trp1 can1 leu2 his3 ura3 rlm1 Δ::kanMX6	this study
YCH246	ade2 trp1 can1 leu2 his3 ura3 PTP2-Myc::HIS3MX6 mpk1 Δ::kanMX6	this study
YCH247	ade2 trp1 can1 leu2 his3 ura3 PTP2-Myc::HIS3MX6 rlm1 Δ::kanMX6	this study
YCH248	ade2 trp1 can1 leu2 his3 URA3::P_{PTP2}-GFP	this study
YCH249	ade2 trp1 can1 leu2 his3 URA3::P_{PTP2}-GFP rlm1 Δ::kanMX6	this study
YCH250	ade2 trp1 can1 leu2 his3 URA3::$P_{PTP2 \Delta 1}$-GFP	this study
YCH251	ade2 trp1 can1 leu2 his3 URA3::$P_{PTP2 \Delta 2}$-GFP	this study
YCH252	ade2 trp1 can1 leu2 his3 URA3::$P_{PTP2 \Delta 1 \Delta 2}$-GFP	this study
YCH253	ade2 trp1 can1 leu2 his3 URA3::P_{PTP2}-PTP2 ptp2 Δ::kanMX6	this study
YCH254	ade2 trp1 can1 leu2 his3 URA3::$P_{PTP2 \Delta 1}$-PTP2 ptp2 Δ::kanMX6	this study
YCH255	ade2 trp1 can1 leu2 his3 URA3::$P_{PTP2 \Delta 2}$-PTP2 ptp2 Δ::kanMX6	this study
YCH256	ade2 trp1 can1 leu2 his3 URA3::$P_{PTP2 \Delta 1 \Delta 2}$-PTP2 ptp2 Δ::kanMX6	this study
YCH257	ade2 trp1 can1 leu2 his3 URA3::P_{ACT1}-PTP2 rlm1 Δ::kanMX6	this study
YCH261	ade2 trp1 can1 leu2 his3 ura3 cmp2 Δ::kanMX6	this study
YCH262	ade2 trp1 can1 leu2 his3 ura3 cna1 Δ::natNT2	this study
YCH263	ade2 trp1 can1 leu2 his3 ura3 cmp2 Δ::kanMX6 cna1 Δ::natNT2	this study
YCH264	ade2 trp1 can1 leu2 his3 ura3 cnb1 Δ::kanMX6	this study
YCH265	ade2 trp1 can1 leu2 his3 ura3 CMP2-Myc::HIS3MX6	this study
YCH266	ade2 trp1 can1 leu2 his3 ura3 CNA1-Myc::HIS3MX6	this study
YCH267	ade2 trp1 can1 leu2 his3 URA3::P_{CMP2}-GFP	this study
YCH268	ade2 trp1 can1 leu2 his3 URA3::P_{CNA1}-GFP	this study
YCH269	ade2 trp1 can1 leu2 his3 URA3::P_{CMP2}-CMP2 cmp2 Δ::kanMX6	this study
YCH270	ade2 trp1 can1 leu2 his3 URA3::P_{CNA1}-CMP2 cmp2 Δ::kanMX6	this study
YCH271	ade2 trp1 can1 leu2 his3 URA3::P_{CNA1}-CNA1 cmp2 Δ::kanMX6	this study
YCH272	ade2 trp1 can1 leu2 his3 URA3::P_{CMP2}-CNA1 cmp2 Δ::kanMX6	this study
YCH281	ade2 trp1 can1 leu2 his3 ura3 mih1 Δ::kanMX6	this study
YCH282	ade2 trp1 can1 leu2 his3 ura3 sdp1 Δ::kanMX6	this study
YCH283	ade2 trp1 can1 leu2 his3 ura3 ych1 Δ::kanMX6	this study
YCH284	ade2 trp1 can1 leu2 his3 ura3 ptc1 Δ::kanMX6	this study
YCH285	ade2 trp1 can1 leu2 his3 ura3 ptc5 Δ::kanMX6	this study
YCH286	ade2 trp1 can1 leu2 his3 ura3 ptc6 Δ::kanMX6	this study
YCH287	ade2 trp1 can1 leu2 his3 ura3 sal6 Δ::kanMX6	this study
YCH288	ade2 trp1 can1 leu2 his3 ura3 ptp1 Δ::kanMX6	this study
YPH499	MAT **a** ura3-52 lys2-801 ade2-101 trp1- Δ63 his3- Δ200 leu2- Δ1	Gift from Dr. Akihiko Nakano
SMY80	MAT **a** ura3-52 lys2-801 ade2-101 trp1- Δ63 his3- Δ200 leu2- Δ1 sec12-4	Gift from Dr. Akihiko Nakano
MBY3-15A	MAT α ura3-52 his3- Δ200 leu2- Δ1 sec13-1	Gift from Dr. Akihiko Nakano
ANS16-1B	MAT α ura3-52 trp1- Δ63 his3- Δ200 leu2- Δ1 sec16-2	Gift from Dr. Akihiko Nakano

10B and YCH are W303 derivatives.

Supplementary Table 3

Family	Gene Name	Forw ard Primer	Reverse Primer
	ACT1	TGCCGAAAGAATGCAAAAGG	TCTGGAGGAGCAATGATCTTGA
	GFP	GGAGAGGGTGAAGGTGATGC	CTTCGGGCATGGCACTCTTG
DSP	*CDC14*	CAACGGTCATGGGGCGCTTA	ACCAGGCGAAGTCTGTGGGA
	MIH1	GAGGGTGCAACGGCAAGATG	TGGACTGGATGACGCAGACG
	MSG5	TTTTCCCGGAGGAGGACTCG	TAGGCGGCAGTACCAGCAAT
	PPS1	CAAACAGAATCAGCGCCAGC	AGCGATCCTCCACTCCCTAC
	SDP1	AGAGCCCACGGTAAAAGAGC	GGTACTCGACAGCAGGAACC
	YCH1	CCAGGTAGTGGATGTGCGAG	TAAGGCGCGAATAGGCGTAG
	YVH1	CCGAGTGTTGAGCCAAACGA	GACCAATTCGCTGCCCGATG
PPP	*CMP2*	TGCAACGGAACTCTTCAGCA	CGATGTAGTGGCCGGATCTC
	CNA1	AGCACACGAAGCACAGGATG	CAGGTAGTTTGGCGCACTGA
	GLC7	AGGGTGATGAGGCCAACAGA	GGCCCTGCAAATCAACTCCA
	PPG1	AGGCGGCCCTGTTCCTGATA	TGACTCGTGGTTTCCCCTCA
	PPH3	TTATGGAGCGACCCCGAAGA	TGCCGACCAGACTGTCACTA
	PPH21	TGGACGTGTTGCAGTTCGAG	CAGGACAAGGGCCACCAATC
	PPH22	CGTGTTGCAGTTCGAGGAGA	GGTGTCAGGACAAGGACCAC
	PPT1	AACGGGATGGGTCCTTCTCA	CTGCTCGAATTGCACACCAC
	PPZ1	TCATGGTGGATTGTCGCCAG	TCCTCCCATTCGTTGGGTGA
	PPZ2	CGTCATTTGGCTCCACTGGG	CGTTGTCTCCGCTATCGTCG
	SAL6	CATCCGCTACGTCTGCTTCC	AAGGCGCACCAAAAGAACCT
	SIT4	GCTTGGCAAGTTTCCCCTCG	AGCCCGGGCAATAAGGTTCA
PPM	*PTC1*	GGATTTAGCCCAACACCAAAG	GCCAGCATACCATTTACACGA
	PTC2	AGGGGACGGGTATTGGATG	ACCTGTTCGTCGTTACATTTGG
	PTC3	ACGGCAACACTTCTTATTTCCA	CCCTTCTTCGCATTTTCATTC
	PTC4	AACCCCACCACAAGAAGCAC	CCCATATACCGTCGCAAGCC
	PTC5	CGGATTTGCCTGAAGTTGCC	AACCATCCGAACCCATCACC
	PTC6	TCTTCCAGGGTACGTGGGTC	GAGCAGTTCAGGGCACTTCC
	PTC7	CCGCGGTAGCTTTCCAACCA	TCCAACACCGTCTGCGACAC
PTP	*PTP1*	AGCATCGAACCGGGCTACTA	TACCAGAGGCGTGACCATCA
	PTP2	TGGATCGCATAGCACAGCA	TTGATTTTGGGGAAGGTTAGACAG
	PTP3	AACCTAAAAGCCCCAAGACAAAC	CGGAGAGGGAGAACTGGAAA
	OCA1	TCACATGCCCCACAAGAACG	ACAGGTGAAGGCTGACCAGA
	LTP1	GGGGAAAGCCCTGATCATCGT	ATGGTCTGCACAGTGCCATC
	SIW14	TGAATCCTGCGAATCAACCGA	GGCCTTCGGAAATGCGAAAC
Other	*PSR1*	GTCGCAGTCGTGGCATTGTA	TGGGGTTGTGGTTGTGGTTG
	PSR2	GGCTACTCCAAACGGCGACA	CCCGCATTGCTGCCTGAATC
	SSU72	AGCTGTGAGACTGCCTGGT	TCGACTTGTAACGGTCTGCTG
